# PRPF19 facilitates colorectal cancer liver metastasis through activation of the Src-YAP1 pathway via K63-linked ubiquitination of MYL9

**DOI:** 10.1038/s41419-023-05776-2

**Published:** 2023-04-08

**Authors:** Rui Zhou, Jie Chen, Yunxiuxiu Xu, Yibiao Ye, Guoping Zhong, Tao Chen, Lin Qiu

**Affiliations:** 1 Department of Hepatobiliary Surgery, Sun Yat-sen Memorial Hospital, Sun Yat-sen University, 510120 Guangzhou, China; 2 Department of Hematology and Oncology, Guangzhou Women and Children’s Medical Center, Guangzhou Medical University, Guangdong Provincial Clinical Research Center for Child Health, Guangzhou, 510623 China

**Keywords:** Colorectal cancer, Metastasis, Ubiquitins

## Abstract

Distant metastasis is one of the leading causes of cancer-related mortality of colorectal cancer (CRC). Dysregulation of E3 ubiquitin ligases has been implicated in acting vital roles in multiple cancers. In this study, we found that the E3 ubiquitin ligase, PRPF19 was positively correlated with liver metastasis, and predicted a worse clinical outcome in CRC. However, the biological effects and the underlying molecular mechanisms of PRPF19 in CRC remain elusive thus far. We illustrated that PRPF19 promoted the migration and invasion capability of CRC cells in both gain- and loss- of function assays. Mechanistically, we uncovered that myosin light chain 9 (MYL9) was the downstream substrate of PRPF19. PRPF19 enhanced the stability of MYL9 via K63-linked ubiquitination, and promoted the migration and invasion capability of CRC cells in an MYL9-mediated manner. Furthermore, the Src–YAP1 cascade was identified as the downstream effector mechanism by which the PRPF19/MYL9 axis promoted metastasis in CRC. Taken together, our findings highlighted that the PRPF19/MYL9 axis served as a novel mechanism in CRC metastasis, which provided an attractive therapeutic strategy for CRC treatment.

## Introduction

Colorectal cancer (CRC) is the third most common type of cancer worldwide, and distant metastasis remains one of the most critical causes of cancer-associated mortality of CRC. The most common metastasis site of CRC is the liver, and nearly 50% of patients with late-stage CRC develop liver metastasis [1, 2]. Therefore, considerable efforts have been concentrated on the relevant mechanisms concerning metastasis of CRC, and searching for novel biomarkers which contribute to improving the outcome of patients with distant metastasis is still an enormous challenge in cancer research.

E3 ubiquitin ligases (E3s) were involved in a variety of cellular processes ranging from protein degradation to modulating protein-protein interactions, from activating to inactivating substrates, and dysregulation of E3s often contributes to cancer development [3–6]. Accumulating evidence revealed that degradation of oncoproteins may contribute to countering the almost inevitable drug resistance due to target inhibition, and increase the peptide presentation capability of MHC molecules which synergize with cancer immunotherapy [5, 7], implying that targeting E3s may provide a promising access for therapeutic intervention of cancer. Pre-mRNA processing factor 19 (PRPF19) is an E3 ligase, involved in regulating cell damage repair [8], inhibits cell cycle arrest and prolongs cell survival in vitro [9, 10]. It is worth mentioning that the expression of PRPF19 is increased in the liver metastasis tumor tissues of CRC in a large-scale proteogenomics study [11], which drew our attention to evaluate the biological behavior of PRPF19 in CRC. As an E3 ligase, the downstream target substrates of PRPF19 are still not well characterized.

Myosin is an actin-dependent molecular motor, which moves along actin filaments and generates mechanical forces within cells, which are transmitted to the extracellular matrix (ECM) through integrin-mediated cell-ECM adhesions, and subsequently lead to ECM remodeling to adapt to changes in mechanical forces [12]. As one component of the actomyosin contractile apparatus, myosin light chain 9 (MYL9) regulates muscle contraction, ECM stiffness, cell shape establishment, and migration through binding to actin filaments to control cytoskeletal dynamics [13], which has been widely identified as a promoter in tumor metastasis in pan-cancer, including CRC [14–17].

Dysregulation of the Hippo pathway components often leads to aberrant cell growth and metastasis in human cancers [18, 19]. YAP1 is a transcription factor downstream of the Hippo pathway, which correlates with a malignant phenotype in cancer [20]. It has been well elucidated that high ECM stiffness could directly lead to the transcriptional activity of YAP1 [21–23]. In the current study, we showed that PRPF19 promoted cell migration and invasion in CRC, and contributed to liver metastasis in CRC patients. Mechanistically, PRPF19 stabilized and activated MYL9 via K63-linked ubiquitination, thereby leading to the activation of Src and YAP1. Our data indicated that PRPF19 may serve as a novel therapeutic target for CRC treatment and diagnostic markers.

## Results

### PRPF19 is upregulated in CRC

**Fig. 1:**
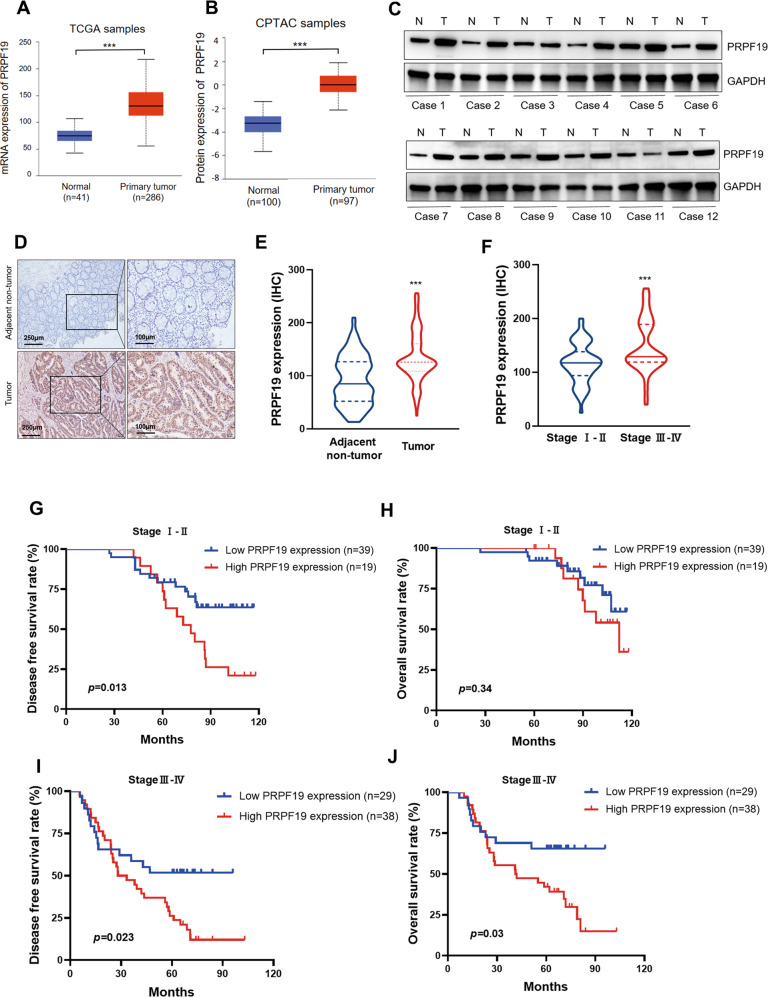
High expression of PRPF19 predicts poor prognosis of patients with CRC. **A**, **B** The mRNA expression (**A**) and protein expression (**B**) of PRPF19 in normal and CRC tissues were evaluated according to the data from TCGA and CPTAC database, respectively. **C** The expression of PRPF19 in 12 pairs of matched adjacent normal tissues (N) and CRC tissues (T). **D**–**F** Representative images of PRPF19 IHC staining in adjacent normal tissues and CRC tissues (**D**), and the expression of PRPF19 in 125 CRC patients were analyzed based on paired Student’s *t* test (**E**) and Student’s *t* test (**F**), respectively. **G**–**J** Kaplan–Meier survival analysis of the association between PRPF19 expression and the DFS or OS rate of CRC patients in stageI-II (**G**, **H**) and stage III–IV (**I**, **J**), respectively.

A previous study has revealed that PRPF19 is upregulated in liver metastasis tumor tissues of CRC in a large-scale proteogenomics study [11], implying that PRPF19 may be a regulator of tumor metastasis in CRC. In this study, we first evaluated the expression level of PRPF19 in various types of cancer, and the data revealed that the expression of PRPF19 was upregulated in pan-cancers (Supplementary Fig. 1A). The mRNA and protein expression levels of PRPF19 were also markedly increased in tumor tissues compared with that in normal tissues in both rectal cancer and colorectal cancer (Fig. 1A, B, Supplementary Fig. 1B). To confirm this issue, we validated the expression levels of PRPF19 in patients’ tumor tissues and CRC cell lines. As shown in Fig. 1C, higher expression of PRPF19 was detected in most of the 12 pairs of CRC tissues compared to the matched adjacent normal tissues, and PRPF19 was also upregulated in most of the CRC cell lines compared with that in normal colon epithelial cell line FHC (Supplementary Fig. 1C). Additionally, the expression of PRPF19 significantly increased in liver metastasis tumor tissues compared with that in paired primary tumor tissues (Supplementary Fig. 1D).

### High expression of PRPF19 predicts poor clinical outcome, and is associated with liver metastasis

Next, we performed immunochemistry (IHC) to determine the prognostic value of PRPF19 in stage I-IV CRC patients, and all the involved patients in stage IV had been diagnosed with liver metastasis. Consistent with the results described above, the expression of PRPF19 dramatically increased in tumor tissues compared with that in paired adjacent non-tumor tissues (Fig. 1D, E), and was higher in tumor tissues of stage III-IV patients than that in stage I-II patients (Fig. 1F).

Subsequently, Kaplan–Meier survival analysis illustrated that high expression of PRPF19 predicted a worse disease-free survival (DFS) and overall survival (OS) rate (Supplementary Fig. 2A, B). To gain further insight into the prognostic value of PRPF19 in patients with metastasis, we also performed survival analysis in stage I-II and stage III-IV patients, respectively. Results indicated that high expression of PRPF19 led to a decreased DFS rate, but had no significant effect on the OS rate of CRC patients in stage I-II (Fig. 1G, H). However, high expression of PRPF19 was inversely associated with both DFS and OS rates for the patients in stage III–IV (Fig. 1I, J).

Furthermore, the correlations between the PRPF19 expression and the clinicopathological features of CRC patients were summarized in Table 1. Results demonstrated that high expression of PRPF19 was significantly associated with the TNM stage (*p* = 0.011), which illustrated that higher expression of PRPF19 indicated a more advanced TNM stage. Moreover, the expression of PRPF19 was also positively correlated with tumor size (*p* = 0.004), tumor invasion (*p* = 0.024), and the presence of lymph nodes (*p* = 0.022) and liver metastasis (*p* = 0.011).

**Table 1:** Correlations between PRPF19 and clinicopathological features.

Variables	Total cases (*n* = 125) (%)	PRPF19 expression		*p* Value
		Low expression (*n* = 68) (%)	High expression (*n* = 57) (%)	
*Gender*				
Male	68 (54.4)	36 (28.8)	32 (25.6)	0.857
Female	57 (45.6)	32 (25.6)	25 (20.0)	
*Age (years)*				
<60	48 (38.4)	26 (20.8)	22 (17.6)	0.556
≥60	77 (61.6)	42 (33.6)	35 (28.0)	
*Size (cm)*				
<5	58 (46.4)	40 (32.0)	18 (14.4)	0.004*
≥5	67 (53.6)	28 (22.4)	39 (31.2)	
*TNM stage*				
I-II	58 (46.4)	39 (31.2)	19 (15.2)	0.011*
III-IV	67 (53.6)	29 (23.2)	38 (30.4)	
*Depth of tumor invasion*				
T1-T2	25 (20.0)	19 (15.2)	6 (4.80)	0.024*
T3-T4	100 (80.0)	49 (39.2)	51 (40.8)	
*Lymph node metastasis*				
N0	58 (46.4)	39 (31.2)	19 (15.2)	0.022*
N1	27 (21.6)	13 (10.4)	14 (11.2)	
N2	40 (32.0)	16 (12.8)	24 (19.2)	
*Liver metastasis*				
M0	87 (69.6)	54 (43.2)	33 (26.4)	0.011*
M1	38 (30.4)	14 (11.2)	24 (19.2)	
*CEA level (ng/ml)*				
<5	63 (50.4)	36 (28.8)	27 (21.6)	0.592
≥5	62 (49.6)	32 (25.6)	30 (24.0)	

*p < 0.05 was considered statistically significant.

To gain further insight into the prognostic value of PRPF19 in patients with CRC, Cox proportional hazard regression analysis was performed to evaluate whether PRPF19 can act as an independent prognostic factor for DFS and OS. Results of univariate and multivariate analyses revealed that the expression of PRPF19, TNM stage, and liver metastasis serve as an independent prognostic factor for DFS and OS in CRC (Tables 2, 3).

**Table 2:** The univariate and multivariate analyses for DFS.

Variables	Univariate analysis		Multivariate analysis	
	Hazard Rratio (95% CI)	*p* value	Hazard ratio (95% CI)	*p* value
Sex (male vs female)	1.40 (0.89–2.20)	0.14		
Age (<60 vs ≥60)	1.21 (0.75–1.94)	0.43		
Tumor size (<5 cm vs ≥5 cm)	1.67 (1.05–2.65)	0.03*		
TNM stage (I-II vs III-IV)	3.21(1.96–5.26)	<0.001*	1.89 (1.03–3.49)	0.04*
Tumor invasion (T1–2 vs T3–4)	1.63 (0.86–3.10)	0.13		
Lymph node metastasis (≤1 vs >1)	3.03 (1.86–4.96)	<0.001*		
liver metastasis (M0 vs M1)	3.82 (2.33–6.28)	<0.001*	2.20 (1.21–4.01)	0.01*
CEA level (<5 ng/ml vs ≥5 ng/ml)	0.94 (0.60–1.49)	0.80		
PRPF19 expression (low vs high)	2.72 (1.70–4.37)	<0.001*	2.19 (1.35–3.56)	0.002*

*p < 0.05 was considered statistically significant.

**Table 3:** The univariate and multivariate analyses for OS.

Variables	Univariate analysis		Multivariate analysis	
	Hazard ratio (95% CI)	*p* value	Hazard ratio (95% CI)	*p* value
Sex (male vs female)	1.39 (0.82–2.38)	0.22		
Age (<60 vs ≥60)	1.20 (0.69–2.10)	0.52		
Tumor size (<5 cm vs ≥5 cm)	1.66 (0.96–2.88)	0.07		
TNM stage (I-II vs III-IV)	6.24 (3.17–12.28)	<0.001*	3.42 (1.51–7.75)	0.003*
Tumor invasion (T1–2 vs T3–4)	1.61 (0.76–3.42)	0.22		
Lymph node metastasis (≤1 vs >1)	4.71 (2.50–8.87)	<0.001*		
liver metastasis (M0 vs M1)	6.87 (3.58–13.20)	<0.001*	3.08 (1.49–6.37)	0.002*
CEA level (<5 ng/ml vs ≥5 ng/ml)	1.03 (0.61–1.76)	0.90		
PRPF19 expression (low vs high)	2.60 (1.49–4.56)	0.01*	1.83 (1.03–3.26)	0.038*

*p < 0.05 was considered statistically significant.

### PRPF19 induces migration and invasion of CRC cells in vitro

**Fig. 2:**
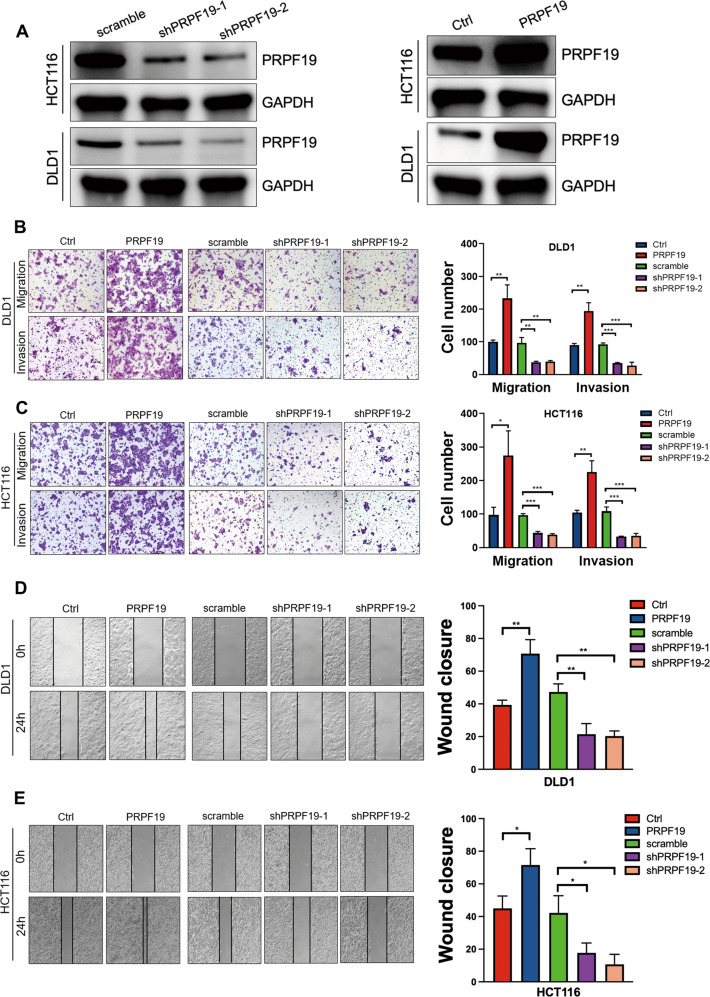
PRPF19 promotes cell mobility in CRC cells. **A** The efficient overexpression and suppression of PRPF19 were confirmed by WB in the indicated stable cell lines. **B**, **C** Transwell assays showed the migration and invasion ability of HCT116 and DLD1 cells with PRPF19 overexpression or knockdown (left panel), and cell quantification was shown (right panel). **D**, **E** Representative images showed the wound healing capability of HCT116 and DLD1 cells with PRPF19 overexpression or knockdown. ***p < 0.001, **p < 0.01, *p < 0.05 based on Student’s *t* test. These experiments were repeated at least three times. Error bars, mean ± SD.

Based on the fact that the basal expression of PRPF19 was the lowest in DLD1 cells, and the highest in HCT116 cells among the seven CRC cell lines (Supplementary Fig. 1C), HCT116, DLD1 were selected to construct the stable cell lines with PRPF19 knockdown (scramble/shPRPF19-1/shPRPF19-2) or overexpression (Ctrl/PRPF19), and the efficiency was validated by Western blot (Fig. 2A, Supplementary Fig. 3A). Subsequently, transwell assays were conducted, and results indicated that upregulation of PRPF19 significantly promoted the migration and invasion capability of CRC cells in DLD1, HCT116, and HCT15 cells, whereas downregulation of PRPF19 caused the opposite effect (Fig. 2B, C, Supplementary Fig. 3B). Similar conclusions were obtained in the wound healing assays (Fig. 2D, E, Supplementary Fig. 3C).

### PRPF19 enhances the stability of MYL9

**Fig. 3:**
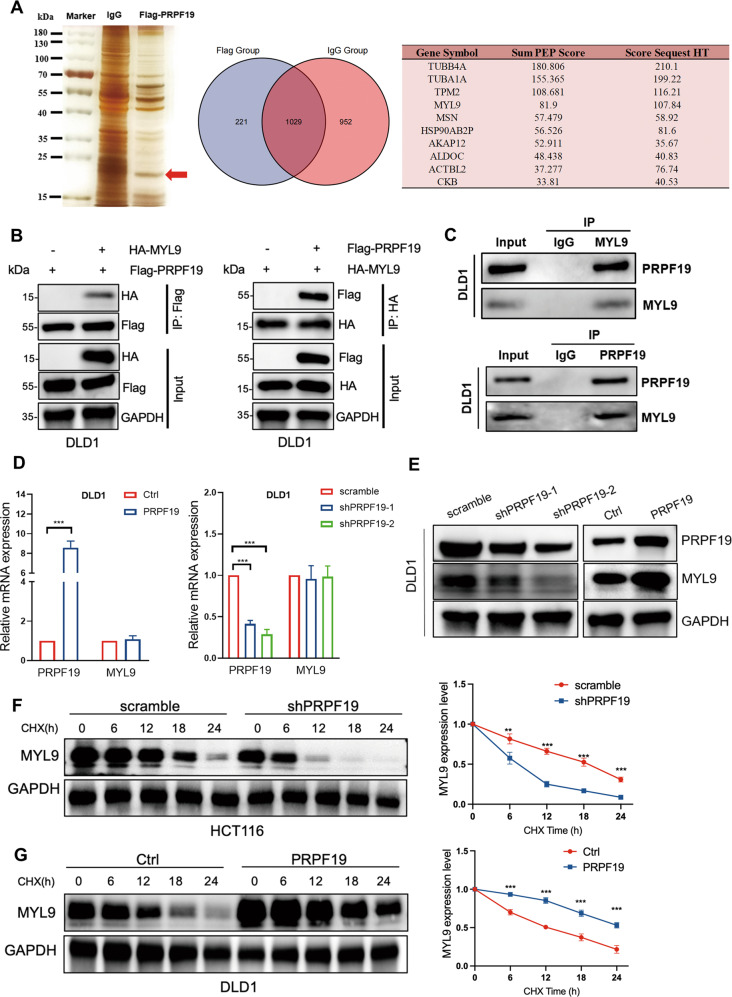
PRPF19 stabilizes MYL9. **A** DLD1 cells were treated with MG132 (20 μM) for 5 h, lysed and subjected to immunoprecipitation with anti-PRPF19 antibody or IgG using silver staining. The red arrow represents the detected bands. The potential interacting proteins of PRPF19 that possessed the highest score were listed (right panel). **B** The DLD1 cells were co-transfected with the indicated plasmids for 48 h, and cells were treated with MG132 for 5 h. The cell lysates were immunoprecipitated with antibody anti-Flag and immunoblotted with anti-HA (left panel); immunoprecipitated with antibody anti-HA and immunoblotted with anti-Flag (right panel). **C** DLD1 cells were treated with MG132 for 5 h before being collected. Cell lysates were immunoprecipitated with anti-IgG, anti-MYL9, or anti-PRPF19 followed by immunoblot. **D**, **E** The mRNA (**D**) and protein (**E**) expression level of MYL9 in the DLD1 stable cell lines. **F**, **G** The indicated stable cell lines with PRPF19 knockdown (scramble/shPRPF19) or overexpression (Ctrl/PRPF19) were treated with CHX, and lysed for WB analysis at the specific time points. The intensity of MYL9 expression for each time point was quantified with GAPDH as a normalizer. ***p < 0.001, **p < 0.01 based on Student’s *t* test.

Given our findings thus far, we determined to elucidate the mechanism by which PRPF19 regulated metastasis in CRC cells. Co-immunoprecipitation (Co-IP) and liquid chromatography-tandem mass spectrometry (LC-MS/MS) were performed to characterize the underlying substrate proteins of PRPF19 following its exogenous expression and immunoprecipitation from DLD1 cells (Fig. 3A). Among the potentially interacted proteins of PRPF19 that possessed the highest score listed in Fig. 3A (right panel), we found that MYL9 was an activator in tumor metastasis and predicted worse outcomes in CRC (Supplementary Fig. 4A, B). Therefore, we emphasized the effect of PRPF19 on the expression of MYL9 in CRC cells.

To demonstrate the exogenous interactions between PRPF19 and MYL9, Flag-PRPF19 and HA-MYL9 were co-transfected into the DLD1 and HCT15 cells, and results of reciprocal co-IP assays showed that PRPF19 indeed interacted with MYL9 (Fig. 3B, Supplementary Fig. 5A). In endogenous IP assays, MYL9 and PRPF19 can be pulled down by each other but not by IgG in both DLD1, HCT15, and HCT116 cells, suggesting that endogenous interactions between MYL9 and PRPF19 (Fig. 3C, Supplementary Fig. 5B, C). In addition, pull-down assays were further performed, and results indicated that PRPF19 directly interacted with MYL9 (Supplementary Fig. 5D).

Next, we evaluated the effect of PRPF19 on the expression level of MYL9 in CRC cells. Results showed that PRPF19 did not alter the mRNA level of MYL9 (Fig. 3D), but led to increased protein expression of MYL9 in DLD1 cells (Fig. 3E), which implied that PRPF19 modulated the expression of MYL9 at the post-transcription level. Likewise, similar results were obtained in HCT15 and HCT116 cells (Supplementary Fig. 5E–H).

Subsequently, cycloheximide (CHX), an inhibitor of protein synthesis, was applied to detect whether PRPF19 affected the stability of endogenous MYL9. As shown in Fig. 3F, G, the knockdown of PRPF19 markedly accelerated the decreased expression of MYL9, while overexpression of PRPF19 protected MYL9 from degradation, which indicated that PRPF19 enhanced the protein stability of MYL9.

### PRPF19 stabilizes MYL9 via inducing K63-linked ubiquitination

**Fig. 4:**
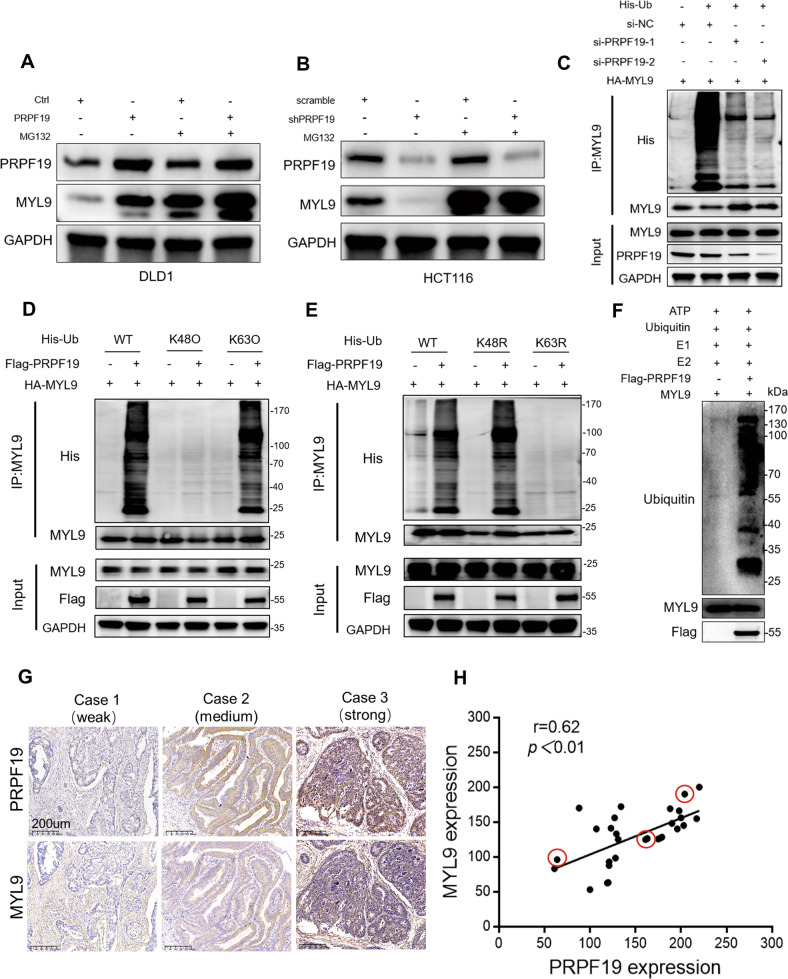
PRPF19 induces K63-linked ubiquitination of MYL9. **A**, **B** The indicated stable cell lines with PRPF19 overexpression (Ctrl/PRPF19) or knockdown (scramble/shPRPF19) were treated with MG132 or DMSO as control. Cell lysates were analyzed by immunoblot. **C** HEK293T cells were transfected with siRNA of PRPF19 for 24 h, and then co-transfected with HA-MYL9 and His-Ub plasmids for another 24 h. After treatment with MG132 for 5 h, cell lysates were collected for co-IP and subjected to immunoblot analysis. **D** In vivo poly-ubiquitination of MYL9. HEK293T cells were co-transfected with plasmids of MYL9, PRPF19 and WT Ub or Ub mutants (K48O, K63O) for 48 h, followed by MG132 treatment for 5 h before lysis. Immunoprecipitation of ubiquitin-conjugated MYL9 protein was performed with anti-His antibody. Immuno-complexes and input were analyzed by immunoblot. **E** HEK293T cells were transfected with the plasmids of MYL9, PRPF19 and WT Ub or Ub mutants (K48R, K63R) for 48 h. Cells were treated with MG132 and lysed for immunoprecipitation and immunoblot as described in (**D**). **F** The in vitro ubiquitination assays were performed and the western blot assays with the indicated antibody. **G**, **H** Representative IHC staining images for PRPF19 and MYL9 of different staining intensities (**G**), and the correlation between the expression of PRPF19 and MYL9 based on the H-score in CRC patient tissues (*n* = 30) were analyzed (**H**).

Since PRPF19 is an E3 ligase, we speculated that MYL9 might be a downstream substrate of PRPF19. We found that the upregulation of MYL9 caused by ectopic expression of PRPF19 could be attenuated by the proteasome inhibitor MG132 in DLD1 and HCT15 cells, while the loss of MYL9 induced by silencing PRPF19 was rescued by MG132 treatment (Fig. 4A, B, Supplementary Fig. 6A), suggesting that PRPF19 protected MYL9 from proteasome degradation. In ubiquitination assays, the knockdown of PRPF19 triggered diminished ubiquitination of MYL9 (Fig. 4C).

K48- and K63-linked ubiquitination are the most common types of ubiquitin linkage types. K48-linked ubiquitination is associated with proteasome degradation, while K63-linked ubiquitination regulates the stabilization of target proteins [24]. To characterize the specific type of MYL9 ubiquitination regulated by PRPF19, plasmids expressing K48-only (containing only K48, substitute arginine for lysine residues at all sites except the indicated one, K48O) or K63-only (K63O) ubiquitin mutants were constructed, and in vivo ubiquitination assays were performed. As shown in Fig. 4D, PRPF19 induced the K63-linked ubiquitination of MYL9. Additionally, K48R and K63R mutants (K48 or K63 lysine mutated to arginine) were also constructed, and results indicated that the K63R mutant dramatically abolished the poly-ubiquitination of MYL9 in the presence of PRPF19, while K48R mutant did not affect the poly-ubiquitination of MYL9 (Fig. 4E). In the in vitro ubiquitination assays, we also confirmed that MYL9 was a direct substrate of PRPF19 (Fig. 4F). These findings illustrated that K63-linked ubiquitination was responsible for the enhanced stability of MYL9 regulated by PRPF19.

Clinically, we examined the expression levels of both PRPF19 and MYL9 in 30 pairs of CRC patient tissues using IHC staining. Results indicated that the expression of PRPF19 was positively correlated with the expression of MYL9 (Fig. 4G, H).

### PRPF19 promotes metastasis in CRC in an MYL9-mediated manner

**Fig. 5:**
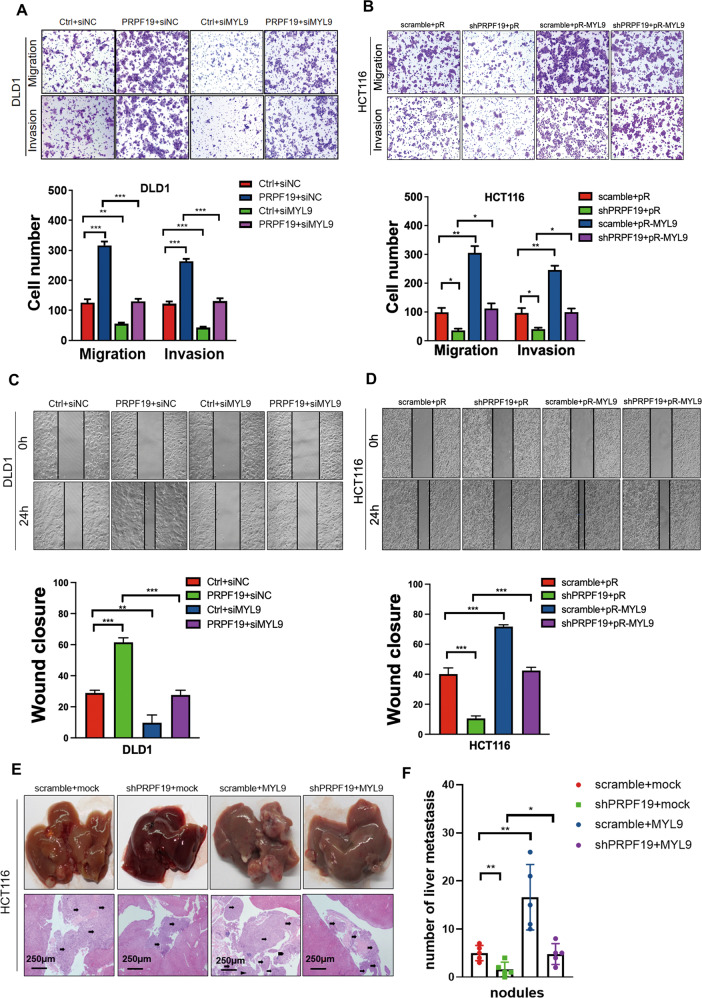
PRPF19 promotes metastasis mediated by MYL9 both in vitro and in vivo. **A**–**D** MYL9 was transiently suppressed by transfecting siRNA into cells, or overexpressed by transfecting plasmids pReceiver-MYL9 (pR-MYL9) or pReceiver (pR) into the indicated cell lines. Representative images of transwell (**A**, **B**) and wound healing (**C**, **D**) assays were shown (upper panel), and quantification was presented (lower panel). The means ± SD of triplicate samples were shown. **E** The livers were collected, and representative images of liver metastatic nodules of HE-stained sections highlighted by arrows were shown. **F** The number of liver metastatic nodules was analyzed. **p < 0.01, *p < 0.05 based on Student’s *t* test.

Based on the findings above, we are curious about the biological function of MYL9 in CRC cells and whether PRPF19 governed the malignant phenotype of CRC via MYL9 activation. Next, MYL9 was transiently knocked down or overexpressed in the stable cell lines with PRPF19 overexpression or knockdown. Consistent with our hypothesis, the knockdown of MYL9 not only substantially suppressed cell mobility, but also abolished the promoting effects on cell migration and invasion of PRPF19 in DLD1 and HCT15 cell lines (Fig. 5A, Supplementary Fig. 6B). Correspondingly, exogenous expression of MYL9 dramatically induced cell migration and invasion in HCT116 cells, and rescued the inhibition effect on cell mobility caused by the knockdown of PRPF19 (Fig. 5B). In accordance with the results above, results in the wound healing assays showed that the effects of PRPF19 on the wound healing abilities of CRC cells could be significantly attenuated by the expression alteration of MYL9 (Fig. 5C, D, Supplementary Fig. 6C).

Anchorage-independent growth is one of the hallmarks of cancer cells, which is associated with the tumorigenic and metastatic potential of cancer cells. Thus, we also further performed sphere formation assays to evaluate the effects of the PRPF19/MYL9 axis on the anchorage-independent growth of CRC cells. Results showed that PRPF19 caused an increased number and size of spheres of DLD1 cells, while the knockdown of MYL9 remarkably reversed the promotion effects of PRPF19. Similar results were also obtained in HCT116 cells (Supplementary Fig. 7). Hence, we concluded that PRPF19 promoted metastasis in CRC in an MYL9-mediated manner.

In addition, hepatic metastasis models were also established to confirm the behavior of the PRPF19/MYL9 axis in liver metastasis in vivo. MYL9 was stably overexpressed in the HCT116 stable cell lines scramble/shPRPF19, and the double stable cell lines were generated, named scramble+mock, shPRPF19+mock, scramble+MYL9, shPRPF19+MYL9, respectively. As shown in Fig. 5E, F, the knockdown of PRPF19 markedly inhibited liver metastasis compared with that in the control group, while MYL9 overexpression significantly rescued the reduced number of liver metastasis nodules caused by the knockdown of PRPF19. Thus, we concluded that PRPF19 promoted liver metastasis of CRC cells via MYL9.

### Src/YAP1 cascade is the downstream effector of the PRPF19/MYL9 axis

**Fig. 6:**
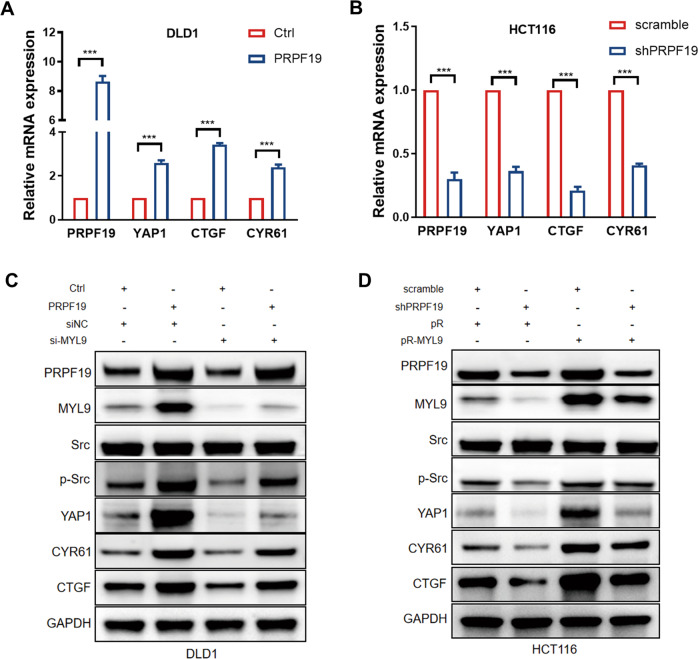
PRPF19/MYL9 axis regulates the Src/YAP1 signaling pathway. **A**, **B** The mRNA expression of YAP1 and its downstream targets were detected by qRT-PCR. **C**, **D** The DLD1 cells stably overexpressed PRPF19 were transfected with siRNA of MYL9, and HCT116 cells stably interfered with PRPF19 were transfected with MYL9 plasmids pReceiver-MYL9 (pR-MYL9) or pReceiver (pR). The indicated molecules were analyzed by WB. ***p < 0.001 based on Student’s *t* test.

**Fig. 7:**
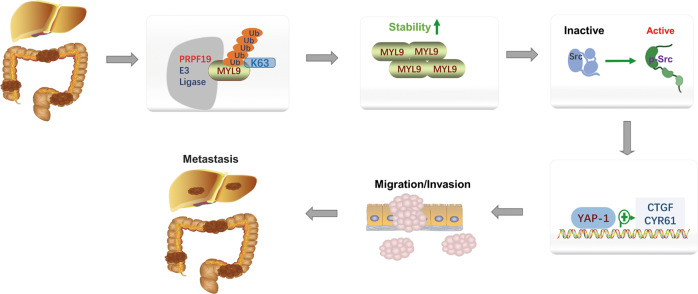
Schematic summary shows that PRPF19 promotes liver metastasis in CRC. PRPF19 enhanced the stability of MYL9 via K63-linked ubiquitination, and the Src-YAP1 pathway acts as the downstream effector mechanism of the PRPF19/MYL9 axis in CRC metastasis.

As a key effector of the Hippo pathway, YAP1 is tightly associated with tumor metastasis. In the process of searching for the underlying downstream mechanisms by which PRPF19 promoted metastasis in CRC, we found that PRPF19 caused the activation of YAP1 and its downstream target genes CTGF, and CYR61 in CRC cells (Fig. 6A, B). Previous studies have proved that myosin and Src functions are required to maintain YAP1 activation in cancer-associated fibroblasts [20], and Src is also an upstream activator of YAP1 in CRC. Therefore, we determined to validate whether the PRPF19/MYL9 axis modulated the expression of Src, YAP1, and the downstream targets of YAP1. Results indicated that overexpression of PRPF19 remarkably led to the accumulation of p-Src, and enhanced the expression of YAP1 and its target genes CTGF, and CYR61, while the knockdown of MYL9 dramatically weakened the effects triggered by PRPF19 (Fig. 6C). Correspondingly, the decreased expression of p-Src and YAP1 caused by the knockdown of PRPF19 could be restored by the exogenous expression of MYL9 (Fig. 6D). In summary, our data demonstrated that PRPF19 promoted metastasis through activating the Src/YAP1 pathway in an MYL9-mediated manner in CRC (Fig. 7).

## Discussion

Dysregulation of the E3s often leads to a variety of diseases, including cancer, making them become attractive drug targets [25]. According to the chain topology, different types of ubiquitination often elicit distinct outcomes. For example, K48-linked ubiquitination is related to the degradation of target proteins, while K63-linked ubiquitination facilitates the activation of target proteins [26]. The final stage of the ubiquitin transfer cascades is catalyzed by E3s, which recruit select targets and thereby determine the substrate specificity. Therefore, targeting E3s is anticipated to yield better specificity and less toxicity. As an E3 ligase, the target substrates of PRPF19 have not been fully understood thus far.

Despite that new treatment approaches have made considerable advances over the past decades, searching for relevant mechanisms concerning distant metastasis is still a formidable challenge in cancer research. We noticed that upregulation of PRPF19 was detected in liver metastasis tumor tissues of CRC in a large-scale proteogenomics study [11], however, there was no report concerning the role of PRPF19 in CRC. Although previous studies have reported that PRPF19 participated in tumorigenesis in several tumor types, its role in cancer is still not well characterized to date [27–29]. Here, we reported that PRPF19 was highly expressed in CRC, and promoted metastasis of CRC cells. Clinically, the expression of PRPF19 was positively correlated with liver metastasis, and predicted an unfavorable prognosis for patients with CRC. However, as an E3 ligase, the target substrates of PRPF19 remain largely unclear thus far.

Tumor metastasis and invasiveness are always initiated and maintained by signaling pathways that control cytoskeletal dynamics and the remodeling of cell-matrix and cell-cell junctions [30]. MYL9, TPM2, and MSN, which are associated with cytoskeletal remodeling and myosin contractility, are all the potential interacted proteins of PRPF19 (Fig. 3A). In the process of searching for the proteins that interacted with PRPF19, we first investigated the prognosis value of MYL9, TPM2 and MSN in CRC. Results revealed that high expression of MYL9 and TPM2 were both significantly associated with a lower OS rate in CRC, while the expression of MSN had no significant effect on the OS rate (Supplementary Fig. 4B–D). Moreover, the expression levels of MYL9 and TPM2, but not MSN were significantly associated with the advanced TNM stage (Supplementary Fig. 4E), implying that MYL9 and TPM2 may play a key role in tumor progression, and predicted a worse outcome in CRC. Subsequently, we validated whether MYL9 and TPM2 were regulated by PRPF19. Results showed that overexpression of PRPF19 induced the protein accumulation of MYL9 (Fig. 3E), while the expression of TPM2 was not significantly regulated by PRPF19. Therefore, we focused on MYL9 for further study.

Tumors are characterized by ECM remodeling and stiffness. ECM stiffness enhances cell growth and promotes migration, and ECM rigidity disrupts tissue morphogenesis by increasing cell tension [20, 21, 30, 31]. Myosin and ECM remodeling are interconnected processes that occur in response to changes in cellular mechanical forces and the mechanical properties of the ECM. Myosin generates mechanical forces within cells, which are transmitted to the ECM, and induced ECM remodeling to adapt to changes in mechanical forces [12]. MYL9 is critically involved in ECM remodeling and stiffness, and induced invasion of tumor cells through binding to actin filaments to control cytoskeletal dynamics [13, 17, 20]. Nevertheless, the regulation mechanism of MYL9 in CRC has not been extensively investigated. We discovered that MYL9 was a downstream target substrate of PRPF19, owing to that PRPF19 stabilized MYL9 by K63-linked ubiquitination, and demonstrated that PRPF19 exerted its pro-metastasis effects in CRC in an MYL9-mediated manner.

Next, we focused on exploring the molecular mechanisms by which the PFPF19/MYL9 axis promoted metastasis in CRC cells. We found that PRPF19 caused the activation of YAP1 and its downstream target genes CTGF, and CYR61 in CRC cells (Fig. 6A, B). It has been well elucidated that high ECM stiffness could directly lead to the transcriptional activity of YAP1 [21–23]. Notably, we noticed that MYL9 plays a critical role in enhancing matrix stiff and invasion of cancer-associated fibroblasts (CAFs), and as the matrix becomes stiffer, isometric tension within the cell increases, leading to Src activation and the subsequent activation of YAP. Since that MYL9 contributes to the stiffer matrix and leads to Src activation, and Src is a well-known upstream driver of YAP in cancer metastasis [32], we supposed that the PRPF19/MYL9 may regulate the Src/YAP pathway in CRC. Consistent with our speculation, results suggested that overexpression of PRPF19 led to the activation of Src, YAP1 and the target genes of YAP1, in an MYL9-mediated manner. Therefore, we hold that the Src/YAP1 cascade functions as one of the key downstream mechanisms by which the PRPF19/MYL9 axis promotes metastasis in CRC.

In summary, our study highlighted the biological function and prognosis value of PRPF19 in CRC. We have illustrated that PRPF19 enhanced the stability of MYL9 through K63-linked ubiquitination, and thereby promoted metastasis via activation of the Src-YAP1 pathway in CRC. A more comprehensive understanding of the role of PRPF19 may provide new access to the therapeutic intervention of CRC.

## Materials and Methods

### Cell culture

The CRC cell lines and HEK293T involved in our study were from American Type Culture Collection (ATCC), and were cultured in appropriate medium containing 10% fetal bovine serum (FBS, Thermo Fisher Scientific, USA) in the incubator at 37 °C in a 5% CO_2_, 95% humidity atmosphere. Mycoplasma contamination was detected by PCR-based method every three months, and cell line authentication was performed using STR DNA profiling.

### Clinical samples and immunohistochemistry

Primary CRC tissues and paired adjacent normal colon epithelium tissues were collected from the surgical specimen of 125 patients clinically and histologically diagnosed with CRC (from 2012 to 2017) by the Sun Yat-sen Memorial Hospital, Sun Yat-sen University, and received no chemotherapy or radiotherapy before surgery. Prior patient consent was obtained for research purposes. Research ethics (SYSKY-2022-170-01) were approved by the Institutional Research Ethics Committee of Sun Yat-sen Memorial Hospital of Sun Yat-sen University, and the study was followed the Declaration of Helsinki. IHC was performed as previously described [33]. The antibodies used were as follows: anti-PRPF19 (#39117, Signalway Antibody Biotech, USA), and anti-MYL9 (#15354, Proteintech, China). The expression intensity was classified as negative = 0, weak = 1, moderate = 2, or strong = 3. The final H score was calculated based on multiplying the intensity score by the percentage of the staining area. A receiver operating characteristic (ROC) curve was generated by plotting the sensitivity and specificity of the H score. Using the cut-off value closest to the maximum sensitivity and specificity, we determined low or high expression of PRPF19.

### SiRNA, plasmids, and cell transfection

SiRNAs were synthesized by the GenePharma Company (Shanghai, China), and the sequences were shown in Supplementary Table S1. Flag-PRPF19 (EX-V1631-M12) and HA-MYL9 plasmids (EX-A4086-M06) were purchased from the GeneCopoeia Company (Guangzhou, China). The His-Ub, His-Ub-K48O, His-Ub-K63O, His-Ub-K48R, and His-Ub-K63R, constructed with pcDNA3.1 were purchased from the Gene Bank (Guangzhou, China). Cells were transfected with the indicated plasmids or siRNA using Lipofectamine 3000 (Thermo Fisher Scientific, USA) following the manufacturer’s instructions.

### RNA extraction and quantitative reverse transcription PCR (qRT-PCR)

Total RNA was isolated from the indicated cells or tissues using Trizol reagent (Thermo Fisher Scientific, USA) following the manufacturer’s instructions. cDNA was synthesized using PrimeScript RT Master Mix (#R323-01, Vazyme, China), and qRT-PCR was performed using a SYBR Green Master Mix (#Q711-02, Vazyme, China). GAPDH expression was used as an endogenous control. The primer used is shown in Supplementary Table S2.

### Western blot and co-immunoprecipitation assays

Western blot was performed as described previously [33]. For co-IP experiments, total cell lysates were obtained using Pierce IP Lysis buffer (87787, Thermo Fisher Scientific, USA) containing protease inhibitor cocktail (P8340, Sigma-Aldrich, USA), and the supernatant was collected. For immunoprecipitation, cell lysates were mixed with the indicated antibody overnight and recovered with Protein A/G Magnetic Beads (HYK0202, MedChem Express, USA). The immunoprecipitates were washed three times, and then analyzed by immunoblot, or silver staining and LC-MS/MS. The antibodies used were summarized in Supplementary Table S3. Silver staining was performed according to the protocol of the Fast Silver Stain Kit (P0017S, Beyotime, China). The LC-MS/MS tests were performed by the Shanghai luming biological technology co.Ltd.

### Stable cell line construction

For stable knockdown of PRPF19, short hairpin RNA (shRNA) was synthesized by the GenePharma Company (Shanghai, China), and the sequences were shown in Supplementary Table S1. Briefly, HEK293T cells were co-transfected with viral packaging plasmids PMD2.G, pSPAX2 and lentiviral constructs (pHBLV-U6-MCS-PGK-PURO) containing the indicated shRNA or control shRNA for 48 h, and virus supernatant was harvested to infect the indicated cells. Puromycin (2 µg/mL) was used for two weeks to select the stably infected cells above.

For stable overexpression of PRPF19 or MYL9, the lentiviral constructs (pHBLV-CMV-MCS-EF1-NEO) containing the indicated cDNA were generated, and were transfected into HEK293T cells along with the viral packaging plasmids PMD2.G and pSPAX2 for 48 h. Cell supernatant was harvested to infect the indicated cells. G418 (0.5 mg/mL) was used to select the stably infected cells for two weeks.

### Cell migration and invasion assays

For the migration assays, transwell inserts (8-mm pore; BD Biosciences, USA) were used to evaluate the migration properties of the indicated cells. 1 × 10^5^ cells suspended in 100 μL serum-free medium were added into the upper side of the inserts membrane, and correspondingly the medium containing 10% FBS was added to the bottom chamber. After 24–48 h of incubation, the cells that did not migrate across the membrane were scraped off, and the cells attached to the lower surface of the inserts were fixed with crystal violet. For the invasion assays, the transwell inserts were coated with Matrigel, and 2 × 10^5^ cells were added as described above. After 24–48 h of incubation, the cells that did not invade across the membrane were erased, and the cells attached to the lower surface of the inserts were fixed. The number of the migrated or invaded cells was evaluated according to the average number of the cells in five randomly selected fields under a microscope at a magnification of 10×. The experiments above were performed in triplicate.

### Wound healing assays

Cells were seeded in 6-well plates (JET BIOFIL, Guangzhou, China) and incubated for 24 h. Subsequently, artificial wounds were scratched with sterile tips, and cells were cultured in medium without FBS. The movement of cells to the scratched area was measured as an indicator of cell migration. Images of wound healing were taken using an inverted microscope at the indicated time point.

### Sphere formation assays

Ultralow attachment plates were used to simulate the matrix detachment environment for anchorage-independent growth of cells. 1 × 10^3^ cells were plated in 24-well ultralow attachment plates with serum-free DMEM/F12 medium (10565018, Gibco) supplemented with 20 ng/ml bFGF (100–18B, PeproTech), 20 ng/ml EGF (AF-100–15, PeproTech), 2% B27 supplement (17504044, Gibco), and 1% penicillin-streptomycin. Fresh serum-free DMEM/F12 medium containing growth factors was supplemented every other day. After 10 days of incubation, spheres greater than 50 μm in diameter per well were counted under a microscope.

### Protein half-life assays

Cells were seeded in 6-well plates for 24 h before being treated with CHX (100 μg/ml, R750107, Sigma-Aldrich), an inhibitor of protein synthesis. Western blots were performed at the indicated time points after cell collection.

### GST pull-down assays

GST-MYL9 and GST proteins were induced and purified from bacteria BL21, and were incubated with the lysates from HEK-293T cells expressing Flag-PRPF19. GST pull-down assay was performed using Pierce GST Protein Interaction Pull-Down Kit (21516, Thermo Scientific) according to the manufacturer’s instructions. The final bound proteins were detected by WB analysis.

### Ubiquitination assays

For in vivo ubiquitination assays, cells were transfected with the indicated plasmids for 48 hours, and then cells were lysed with proteasome inhibitor MG132 (20 μM, HY13259, MedChem Express, USA) for 5 h. Cell lysates were prepared and incubated overnight with an anti-MYL9 antibody. Protein A/G beads were then added and incubated for 2 h. Protein A/G beads were washed with IP wash buffer three times, and the immunoprecipitates were detected by Western blot.

For in vitro ubiquitination assays, MgATP Solution (R&D Systems, B-20), UBE1 (E1) (E-305-025, R&D Systems), UbcH5c/UBE2D3 (E2) (E2-627-100, R&D Systems), PRPF19 protein (Self-laboratory purification), MYL9 protein (Proteintech, Ag7636), 10X E3 Ligase Reaction Buffer (B-71, R&D Systems), Ubiquitin Recombinant Human HA-Ubiquitin Protein (U-110-01M, R&D Systems) and ddH20 were compounded in a 25ul reaction system following the manufacturer’s instructions. The reaction mixture was incubated at 37 °C for 60 min. Reactions were stopped by SDS buffer and resolved by SDS–PAGE for Western blot.

### Animal experiments

All animal experiments were followed the ARRIVE guidelines. The Animal Care and Use Committee of Guangzhou Medical University approved the study (S2022-100). We purchased male BALB/c nude mice from Guangdong Medical Laboratory Animal Center and randomly assigned the mice to four groups. 2 × 10 ^6^ HCT116 cells suspended in 100 μL PBS were injected into the distal tip of the spleen. Mice were euthanized eight weeks after injection, and livers were harvested to examine the metastatic nodules under a microscope. Transverse sections were stained with H&E for histology analysis.

### Bioinformatics analysis

The Cancer Genome Atlas (TCGA) and Clinical Proteomic Tumor Analysis Consortium (CPTAC) databases were used to obtain clinical data of CRC patients and gene copy number variations or protein expression. Kaplan–Meier survival analysis of MYL9, TPM2, MSN in CRC were completed based on the data from the GEPIA database (http://gepia.cancer-pku.cn/). UALCAN (http://ualcan.path.uab.edu/index.html) [34, 35], an interactive web portal, which performs in-depth analyses of TCGA, was used to compare the expression of PRPF19 in CRC tissues with that in normal colon epithelium tissues.

### Statistical analysis

The statistical analysis was performed using GraphPad Prism 8.0 (GraphPad Software, USA). Data was represented as mean ± SD. The two-tailed *t*-test was used to compare two independent groups, and the two-tailed χ 2 test was applied to evaluate the correlations between PRPF19 expression and clinicopathological features. Log-rank tests were used to compare Kaplan–Meier survival curves. An analysis of univariate and multivariate variables was conducted using Cox proportional hazard regression models. For correlation analysis, Pearson’s test was applied. *p* ≤ 0.05 was considered statistically significant.

## Supplementary information

Supplementary Figure legend

Supplementary Figure 1

Supplementary Figure 2

Supplementary Figure 3

Supplementary Figure 4

Supplementary Figure 5

Supplementary Figure 6

Supplementary Figure 7

Supplementary Table S1

Supplementary Table S2

Supplementary Table S3

checklist

Original Data File

## Data Availability

All data generated or analyzed during this study are included in this published article and its supplementary information files.
