# Supplementary material for: PRPF19 facilitates colorectal cancer liver metastasis through activation of the Src-YAP1 pathway via K63-linked ubiquitination of MYL9
Source: Cell Death Dis. 2023 Apr 8;14(4):258. doi: 10.1038/s41419-023-05776-2 (PMC10082770; doi:10.1038/s41419-023-05776-2)
Supplement: Supplementary file 1 — Supplementary Figure legend [file 41419_2023_5776_MOESM1_ESM.docx]

**Supplementary Figure 1**

**(A)** The expression level of PRPF19 in pan-cancers according to the data from CPTAC datasets. **(B)** The mRNA expression of PRPF19 in normal (N) and CRC tissues (T) was evaluated in both colorectal cancer and [rectal cancer](javascript:;) according to the data from TCGA. **(C)** Immunoblots of PRPF19 expression in normal colon epithelial cell line FHC and seven CRC cell lines. **(D)** The mRNA expression of PRPF19 in 24 pairs of primary tumor tissues and matched liver metastatic tumor tissues.

**Supplementary Figure 2**

**(A-B)** Kaplan–Meier survival analysis of the association between PRPF19 expression and DFS or OS in collected CRC patient tissues.

**Supplementary Figure 3**

**(A)** The efficient overexpression of PRPF19 was confirmed by WB. **(B)** Transwell assays showed the migration and invasion ability of HCT15 cells with PRPF19 overexpression (left panel), and cell quantification was shown (right panel). **(C)** Representative images showed the wound healing capability of HCT15 cells with PRPF19 overexpression. ***, *p*<0.001, **, *p*<0.01, based on Student’s *t*-test. These experiments were repeated at least three times. Error bars, mean ± SD.

**Supplementary Figure 4**

**(A-D)** Kaplan–Meier curves showing the DFS or OS rate based on the expression of MYL9 (A-B), TPM2(C), MSN(D) in CRC tissues from the GEPIA database.

**Supplementary Figure 5**

(A) Cells were co-transfected with the indicated plasmids for 48 h, and were treated with MG132 for 5 h. The cell lysates were immunoprecipitated with antibody anti-Flag and immunoblotted with anti-HA (left panel); immunoprecipitated with antibody anti-HA and immunoblotted with anti-Flag (right panel). **(B-C)** The indicated cells were treated with MG132 for 5 h before being collected. Cell lysates were immunoprecipitated with anti-IgG, anti-MYL9, or anti-PRPF19 followed by immunoblot. **(D**) MYL9 directly binds to PRPF19 in vitro. GST or GST- MYL9 was incubated with the lysates from HEK293T cells expressing Flag-PRPF19, and the bound proteins were analyzed with anti-Flag antibody. **(E-H)** The mRNA and protein expression level of MYL9 in the indicated HCT116 and HCT15 stable cell lines.

**Supplementary Figure 6**

**(A)** The HCT15 stable cell lines with PRPF19 overexpression (Ctrl/PRPF19) were treated with MG132 or DMSO as control. Cell lysates were analyzed by immunoblot. **(B-C)** MYL9 was transiently suppressed in the HCT15 stable cell lines with PRPF19 overexpression (Ctrl/PRPF19). Representative images of transwell (B) and wound healing (C) assays were shown (left panel), and quantification was presented (right panel). The means ± SD of triplicate samples were shown.

**Supplementary Figure 7**

**(A-B)** MYL9 was transiently suppressed or overexpressed in the indicated stable cell lines. Representative images of sphere formation assays were shown (left panel), and quantitative data was presented (right panel). The means ± SD of triplicate samples were shown.
