# Supplementary figures and images for: PRPF19 facilitates colorectal cancer liver metastasis through activation of the Src-YAP1 pathway via K63-linked ubiquitination of MYL9
Source: Cell Death Dis. 2023 Apr 8;14(4):258. doi: 10.1038/s41419-023-05776-2 (PMC10082770; doi:10.1038/s41419-023-05776-2)

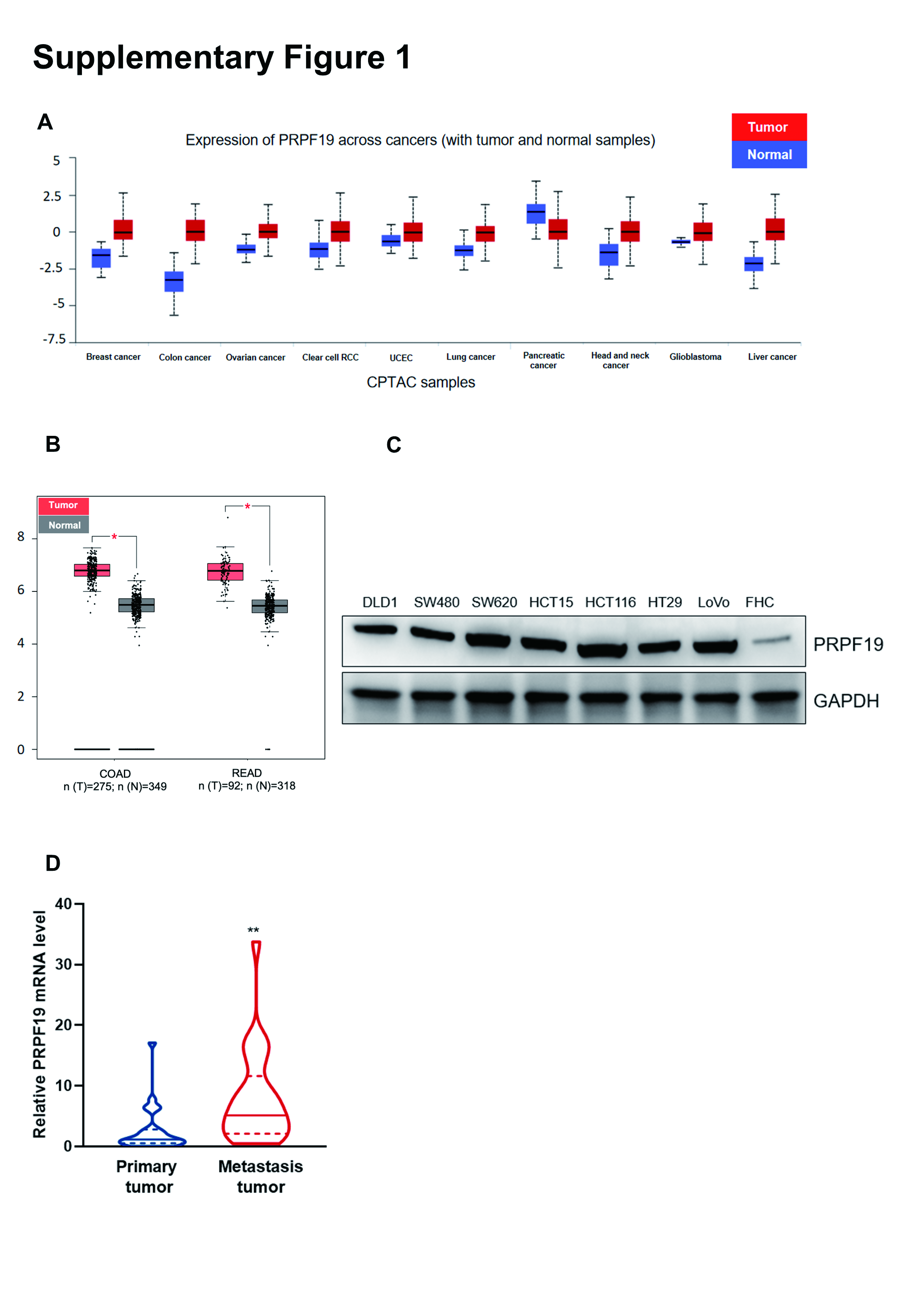

Supplement: Supplementary file 2 — Supplementary Figure 1 [file 41419_2023_5776_MOESM2_ESM.tif]

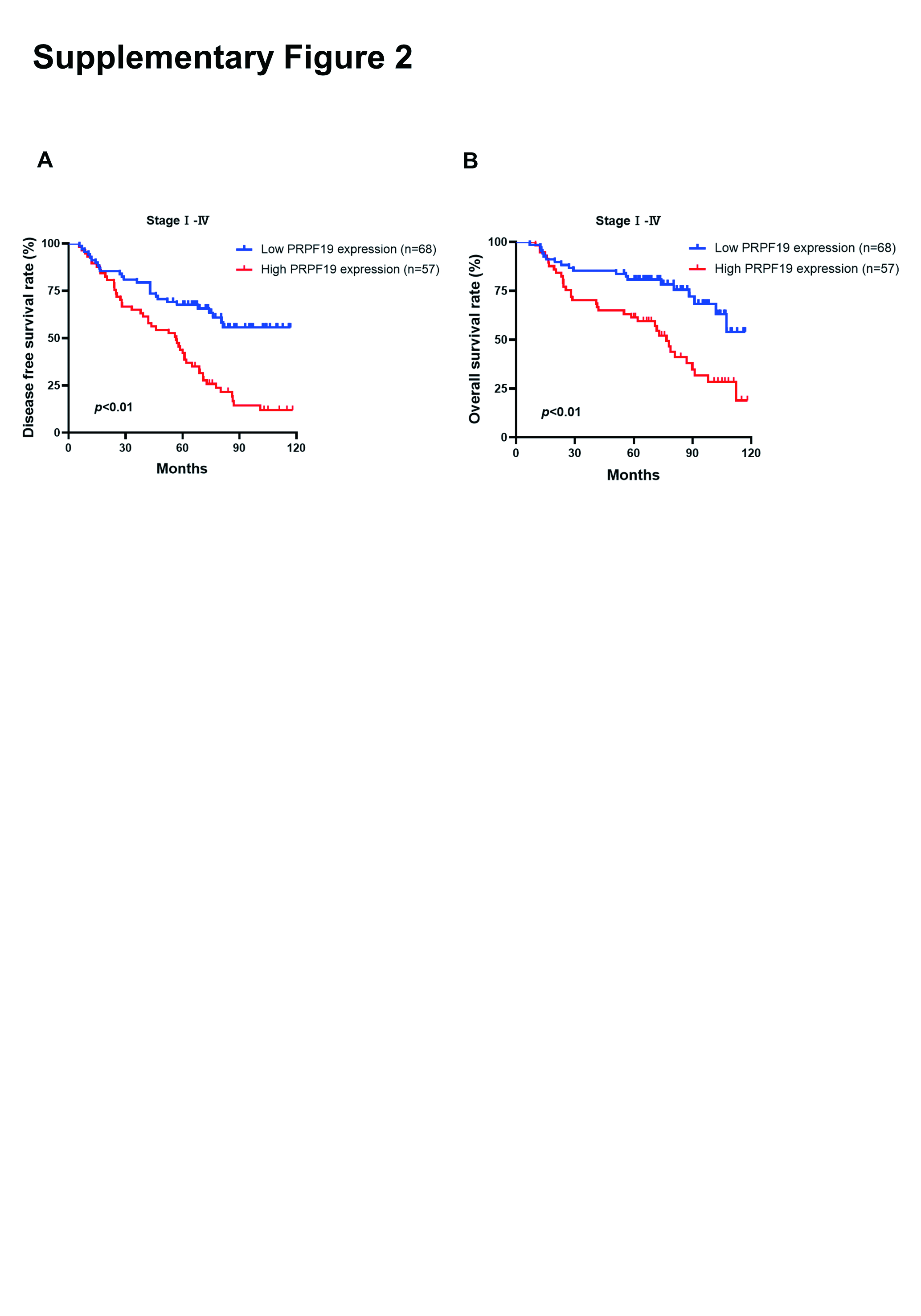

Supplement: Supplementary file 3 — Supplementary Figure 2 [file 41419_2023_5776_MOESM3_ESM.tif]

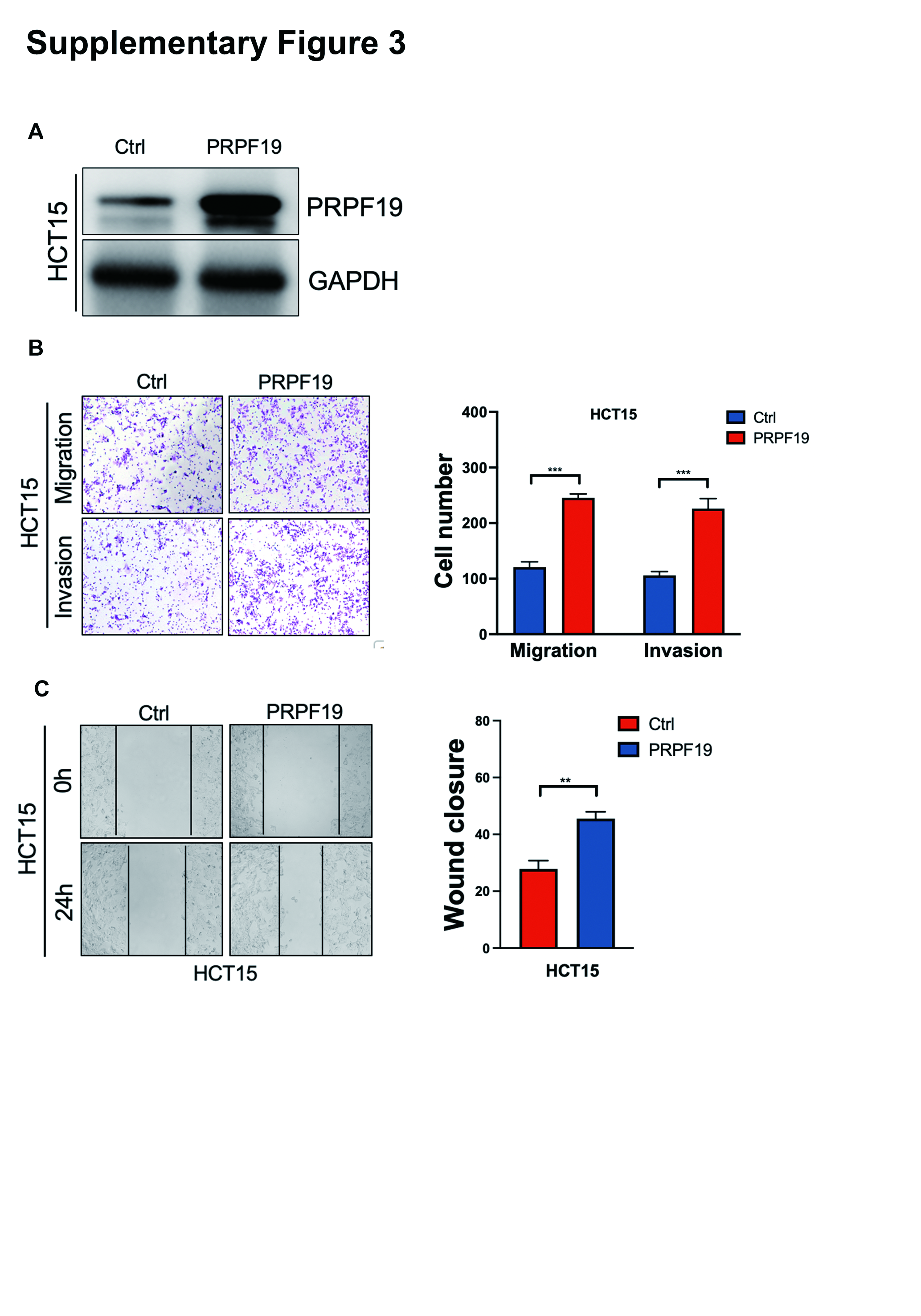

Supplement: Supplementary file 4 — Supplementary Figure 3 [file 41419_2023_5776_MOESM4_ESM.tif]

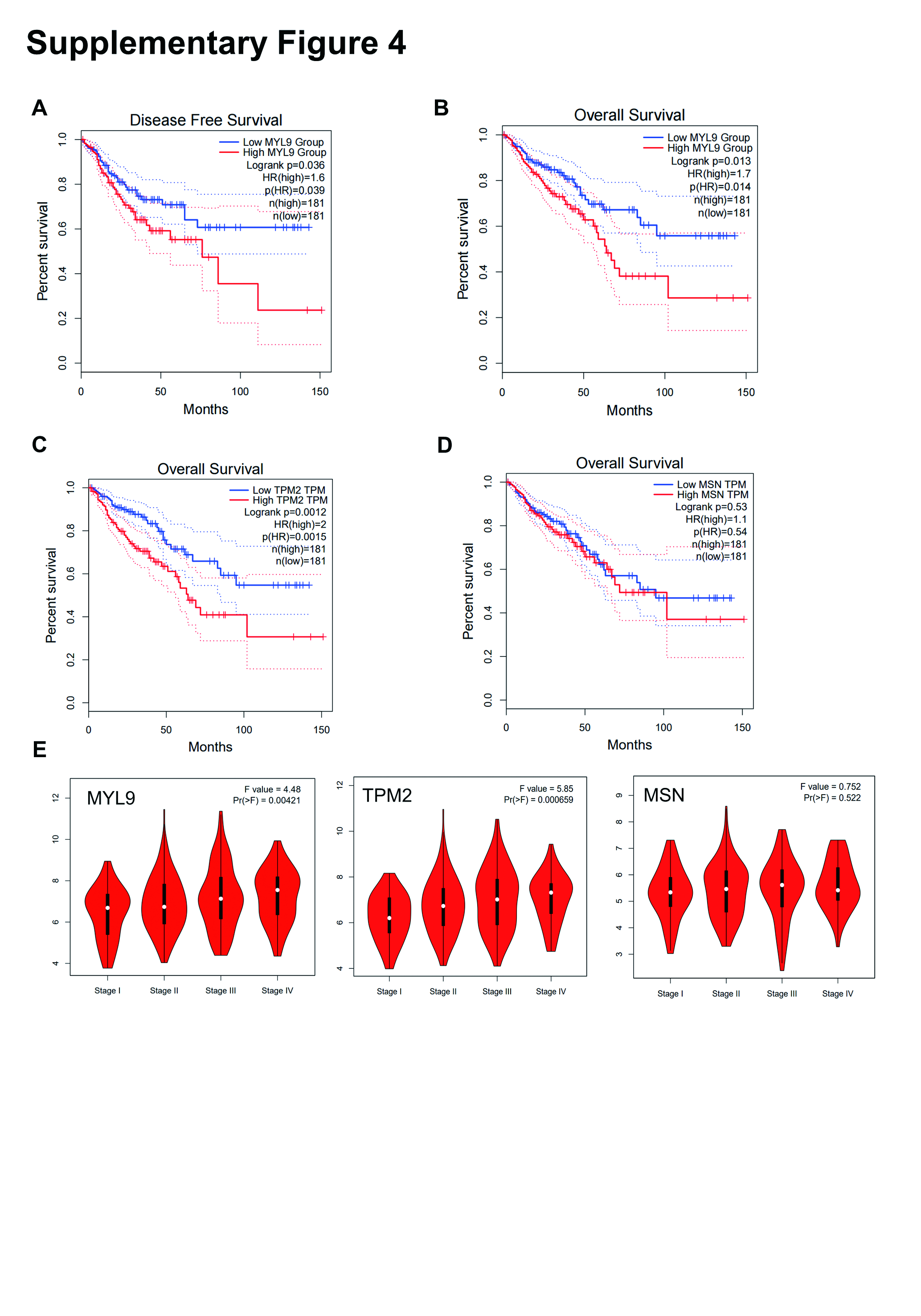

Supplement: Supplementary file 5 — Supplementary Figure 4 [file 41419_2023_5776_MOESM5_ESM.tif]

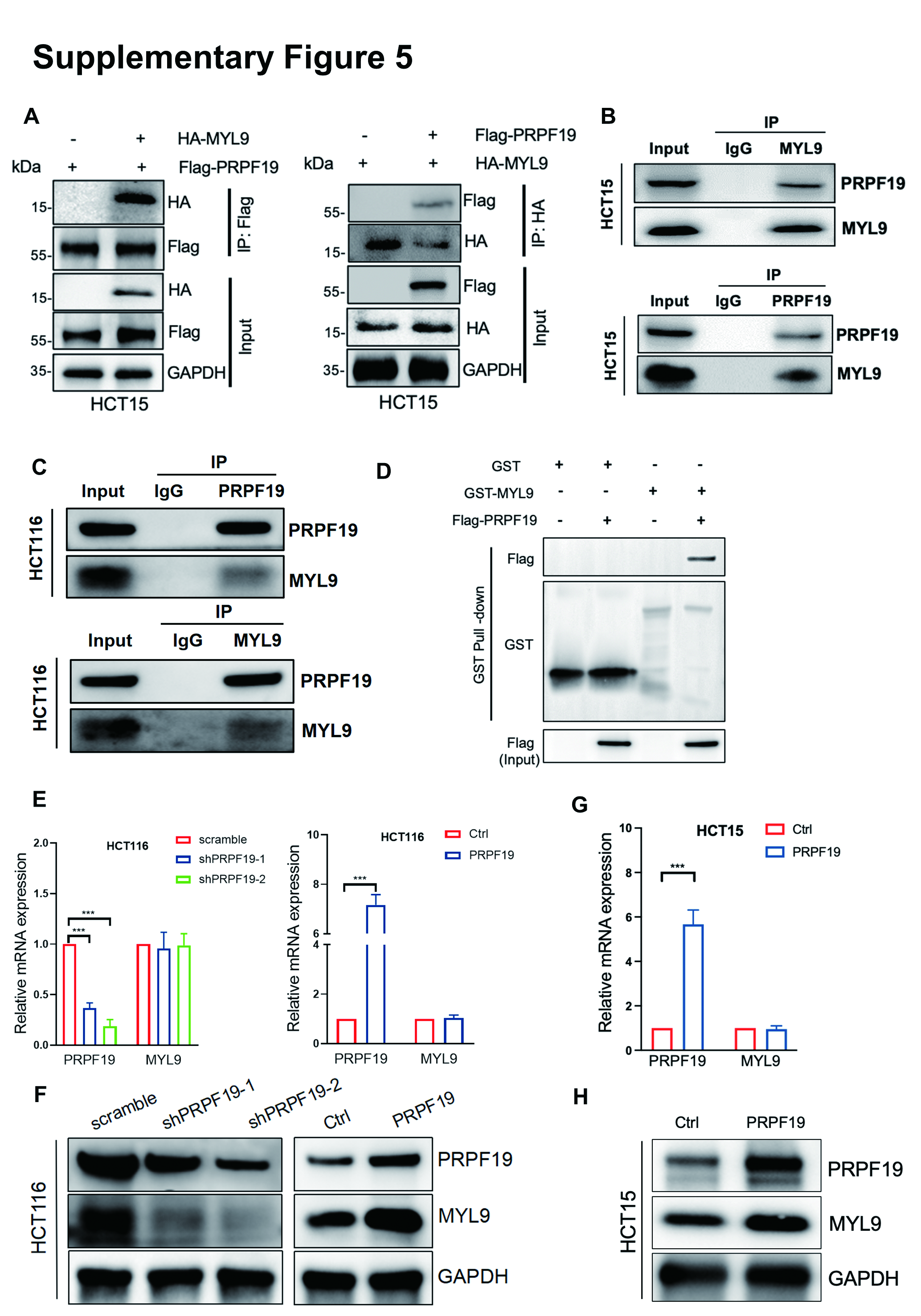

Supplement: Supplementary file 6 — Supplementary Figure 5 [file 41419_2023_5776_MOESM6_ESM.tif]

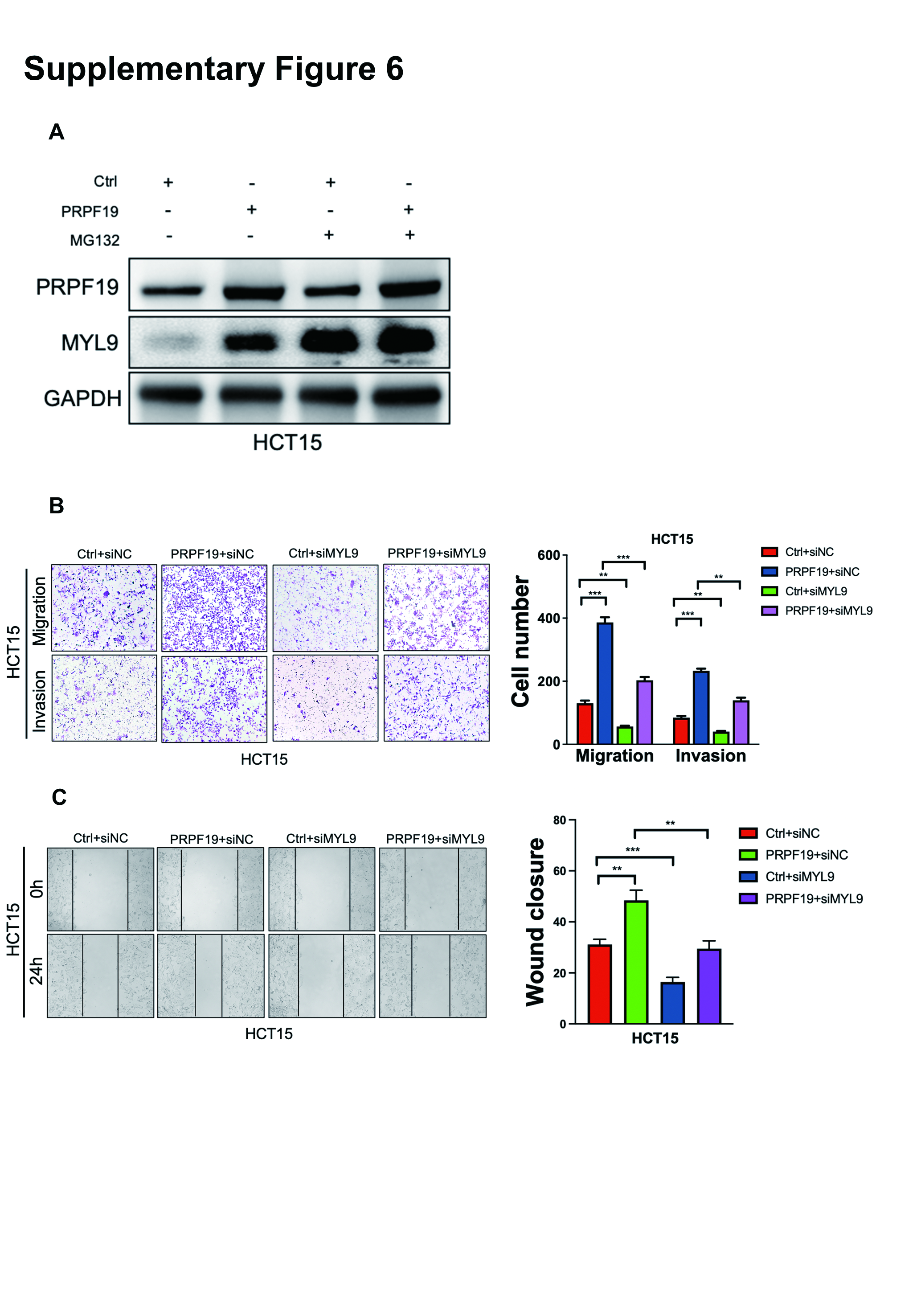

Supplement: Supplementary file 7 — Supplementary Figure 6 [file 41419_2023_5776_MOESM7_ESM.tif]

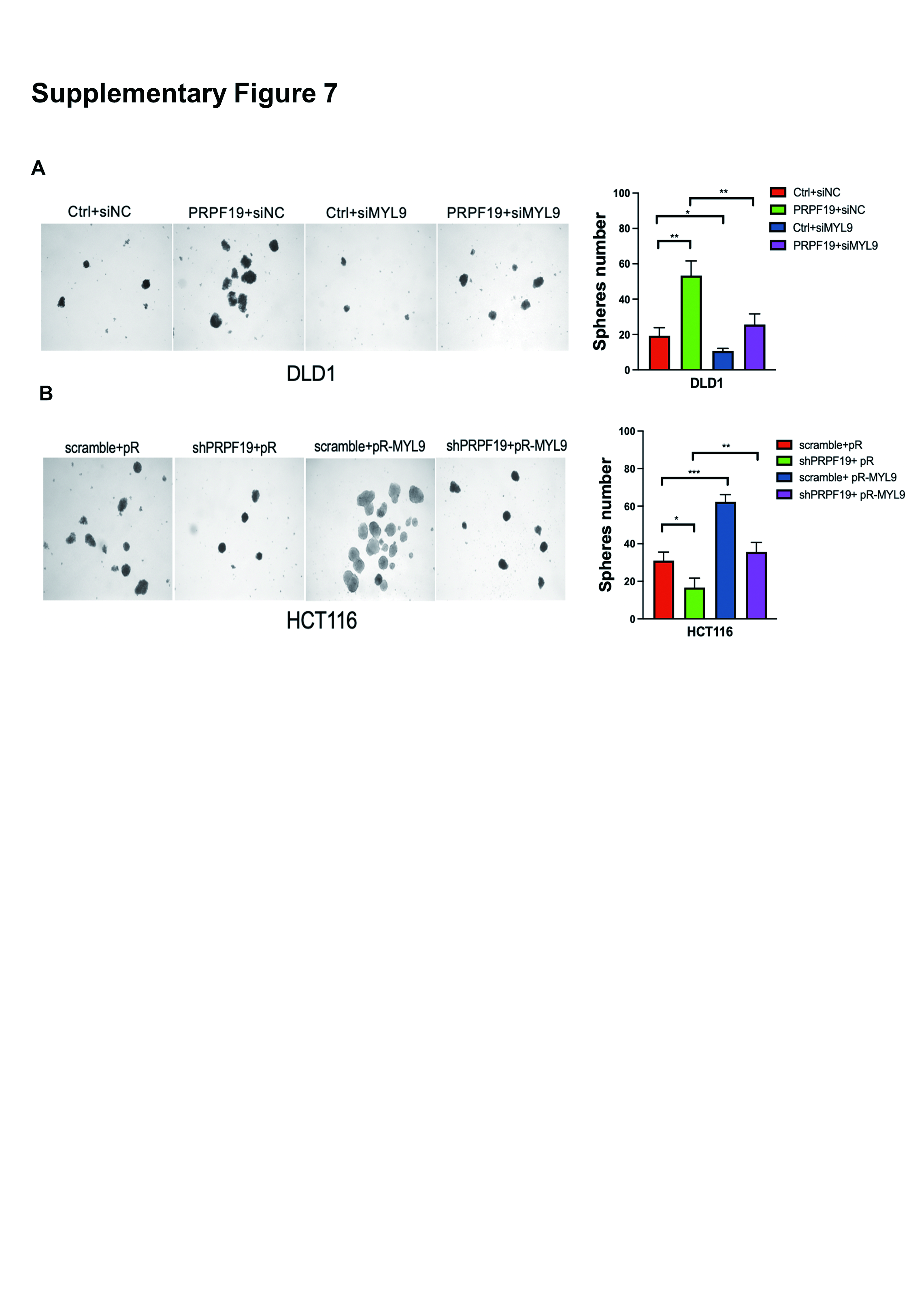

Supplement: Supplementary file 8 — Supplementary Figure 7 [file 41419_2023_5776_MOESM8_ESM.tif]
