# Supplementary material for: PRPF19 facilitates colorectal cancer liver metastasis through activation of the Src-YAP1 pathway via K63-linked ubiquitination of MYL9
Source: Cell Death Dis. 2023 Apr 8;14(4):258. doi: 10.1038/s41419-023-05776-2 (PMC10082770; doi:10.1038/s41419-023-05776-2)
Supplement: Supplementary file 9 — Supplementary Table S1 [file 41419_2023_5776_MOESM9_ESM.docx]

**Supplementary Table S1**

**SiRNA sequences**

| **Gene** | **Oligonucleotide** |
| --- | --- |
| si-PRPF19-1 | GAUGGAAGCUGGGCCUCAUCUCAGUdTdT |
| si-PRPF19-2 | CCUGUCUCUAAUCAUGUUUAUdTdT |
| siNC | CGUACGCGGAAUACUUCGAdTdT |
| siMYL9 | CCAAGGAUAAAGACGACUAdTdT |

**ShRNA sequences**

| **Gene** | **Oligonucleotide** |
| --- | --- |
| shPRPF19-1 | GATGGAAGCTGGGCCTCATCTCAGT |
| shPRPF19-2 (shPRPF19) | CCTGTCTCTAATCATGTTTAT |
