# Supplementary material for: PRPF19 facilitates colorectal cancer liver metastasis through activation of the Src-YAP1 pathway via K63-linked ubiquitination of MYL9
Source: Cell Death Dis. 2023 Apr 8;14(4):258. doi: 10.1038/s41419-023-05776-2 (PMC10082770; doi:10.1038/s41419-023-05776-2)
Supplement: Supplementary file 10 — Supplementary Table S2 [file 41419_2023_5776_MOESM10_ESM.docx]

**Supplementary Table S2**

**Primer sequences for qRT–PCR.**

| **Gene** | **Sense primer** | **Antisense primer** |
| --- | --- | --- |
| PRPF19 | GTGCCAAGTTCCCAACCAAGTGTT | AGCACAGTGGCTTTGTCTTGAAGC |
| MYL9 | GGACCCCGAGGATGTGATTC | TTGAGGATGCGGGTGAACTC |
| YAP1 | TAGCCCTGCGTAGCCAGTTA | TCATGCTTAGTCCACTGTCTGT |
| CTGF | ACCGACTGGAAGACACGTTTG | CCAGGTCAGCTTCGCAAGG |
| CYR61 | AGCCTCGCATCCTATACAACC | TTCTTTCACAAGGCGGCACTC |
| GAPDH | ACGGATTTGGTCGTATTGGGC | TTGACGGTGCCATGGAATTTG |
