# Supplementary material for: PRPF19 facilitates colorectal cancer liver metastasis through activation of the Src-YAP1 pathway via K63-linked ubiquitination of MYL9
Source: Cell Death Dis. 2023 Apr 8;14(4):258. doi: 10.1038/s41419-023-05776-2 (PMC10082770; doi:10.1038/s41419-023-05776-2)
Supplement: Supplementary file 11 — Supplementary Table S3 [file 41419_2023_5776_MOESM11_ESM.docx]

**Supplementary Table S3**

**Antibodies information**

| **Antibody** | **Source** | **Article Number** |
| --- | --- | --- |
| PRPF19 | Signalway Antibody Biotech | 39117 |
| MYL9 | Proteintech | 15354 |
| HA-Tag | Cell Signaling Technology | 3724 |
| His-Tag | Cell Signaling Technology | 12698 |
| Flag-Tag | Cell Signaling Technology | 14793 |
| GST-Tag | Proteintech | 10000-0-AP |
| YAP1 | HUABIO | ET1608-30 |
| CTGF | Cell Signaling Technology | 86641 |
| CYR61 | Cell Signaling Technology | 14479 |
| Src | Cell Signaling Technology | 2109 |
| p-Src | Cell Signaling Technology | 2101 |
| GAPDH | Proteintech | 10494 |
| Goat Anti-Rabbit IgG | Abcam | Ab6721 |
| Ubiquitin | HUABIO | ER31212 |
