# Supplementary material for: PRPF19 facilitates colorectal cancer liver metastasis through activation of the Src-YAP1 pathway via K63-linked ubiquitination of MYL9
Source: Cell Death Dis. 2023 Apr 8;14(4):258. doi: 10.1038/s41419-023-05776-2 (PMC10082770; doi:10.1038/s41419-023-05776-2)

Figure 1C

PRPF19

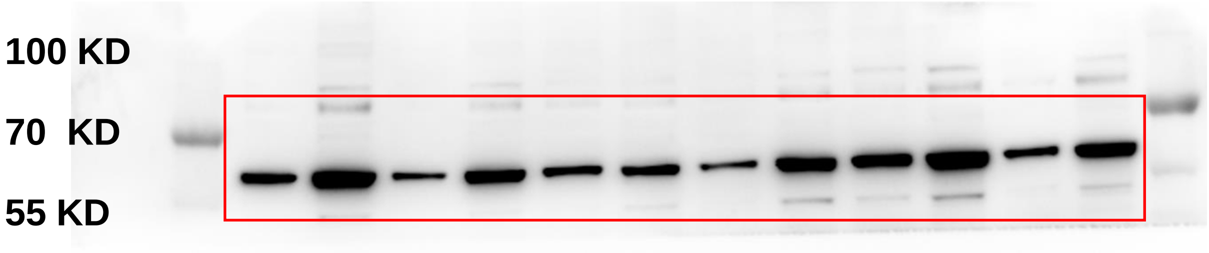

GAPDH

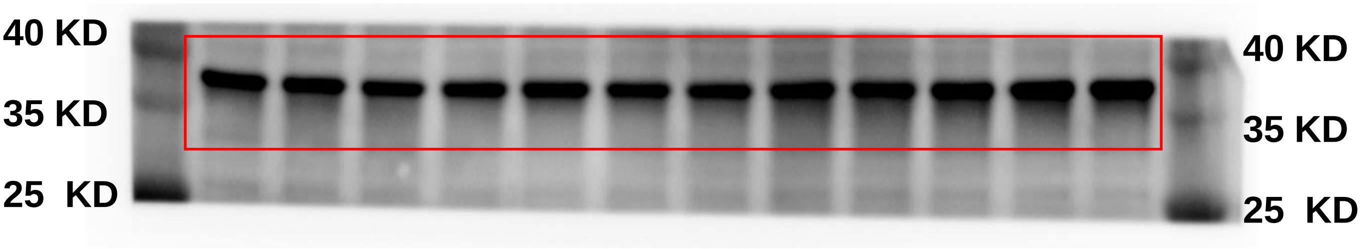

PRPF19

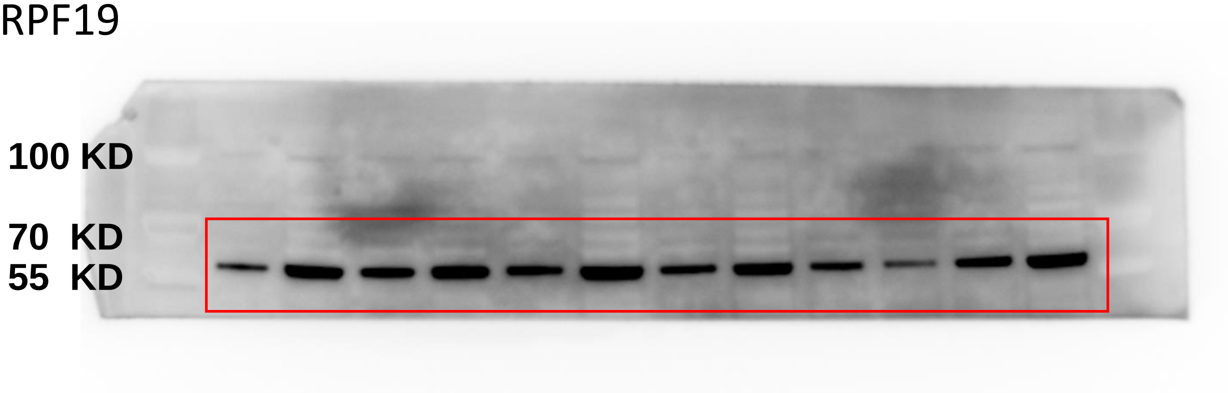

GAPDH

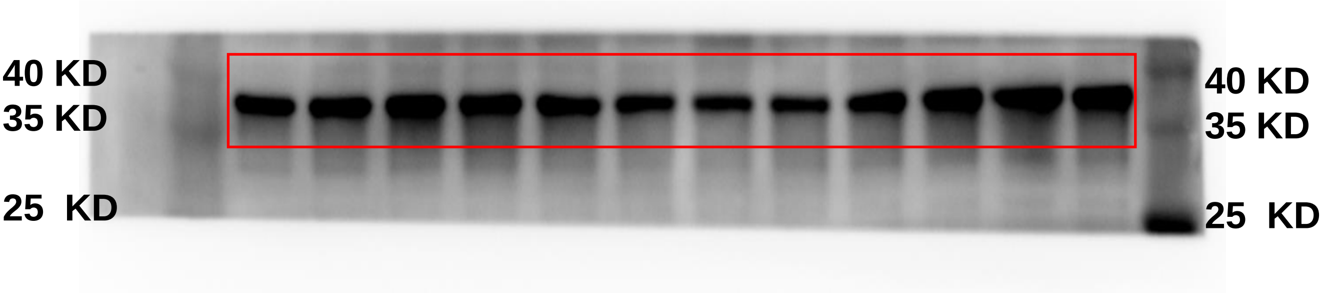

Figure 2A

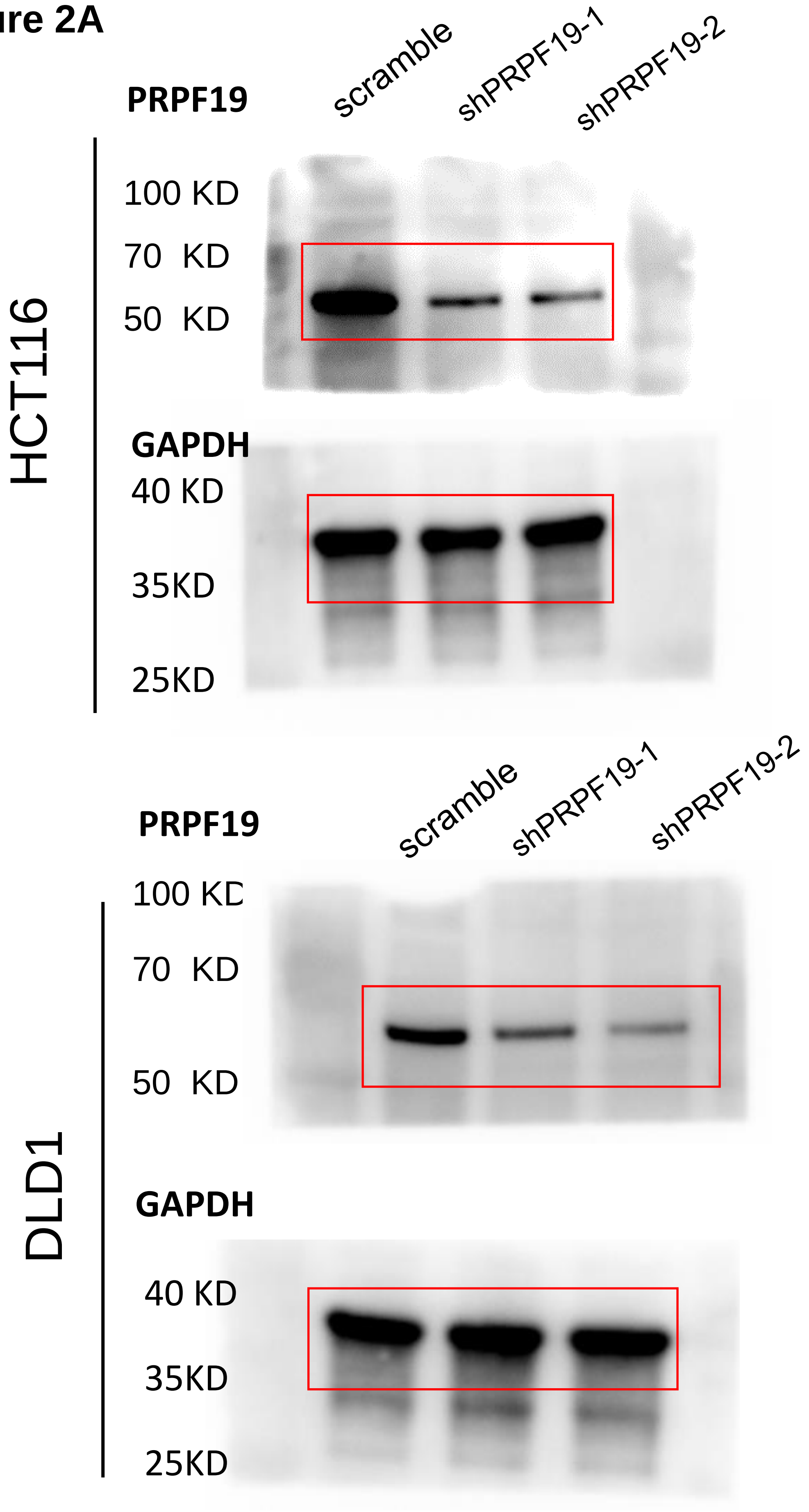

HCT116
PRPF19
scramble
shPRPF19-1
shPRPF19-2
100 KD
70 KD
50 KD
GAPDH
40 KD
35KD
25KD
DLD1
PRPF19
scramble
shPRPF19-1
shPRPF19-2
100 KD
70 KD
50 KD
GAPDH
40 KD
35KD
25KD

Figure 2A

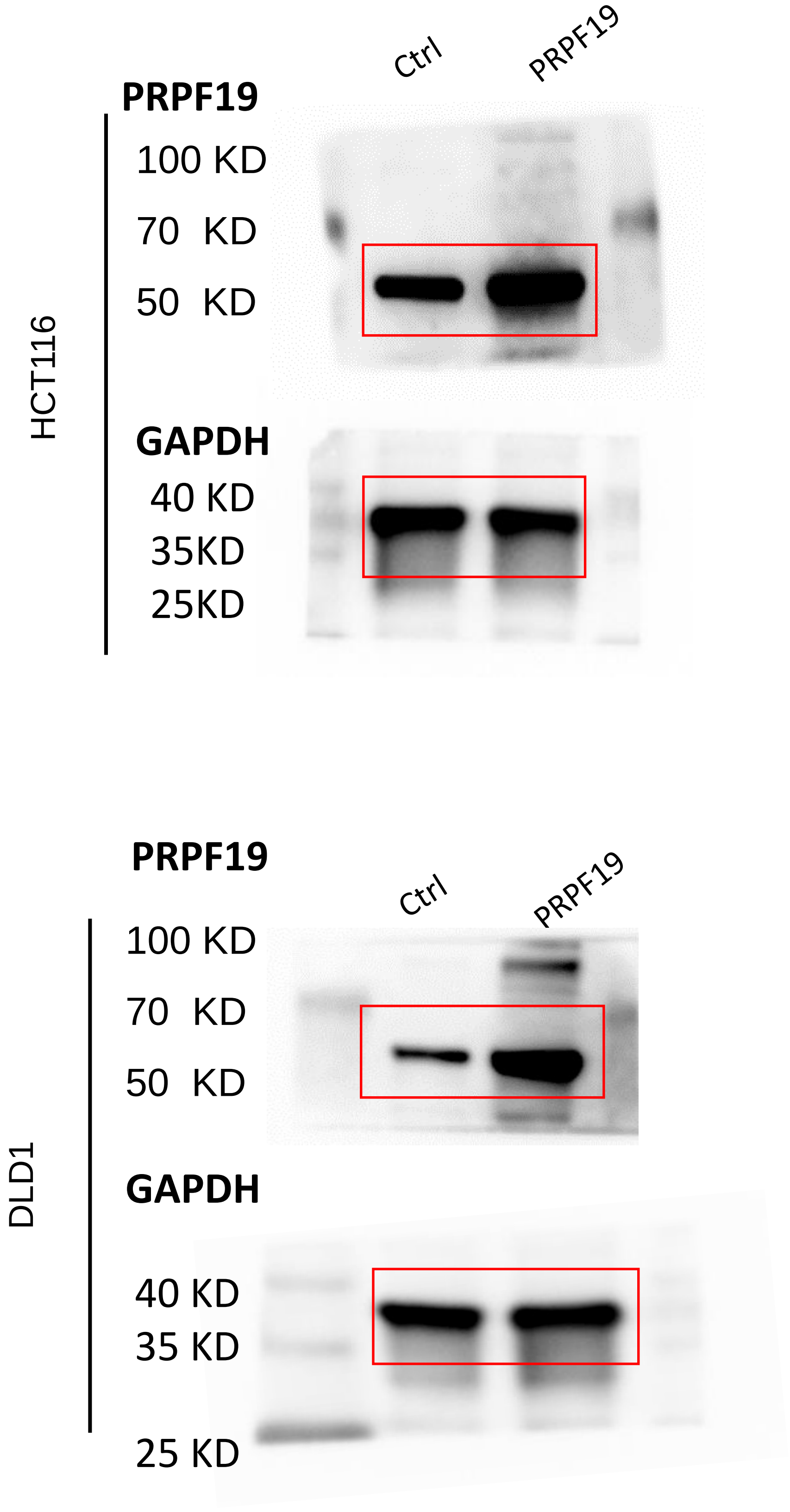
Ctrl
PRPF19
PRPF19
100 KD
70 KD
50 KD
HCT116
GAPDH
40 KD
35KD
25KD
PRPF19
Ctrl
PRPF19
100 KD
70 KD
50 KD
DLD1
GAPDH
40 KD
35 KD
25 KD

Figure 3B

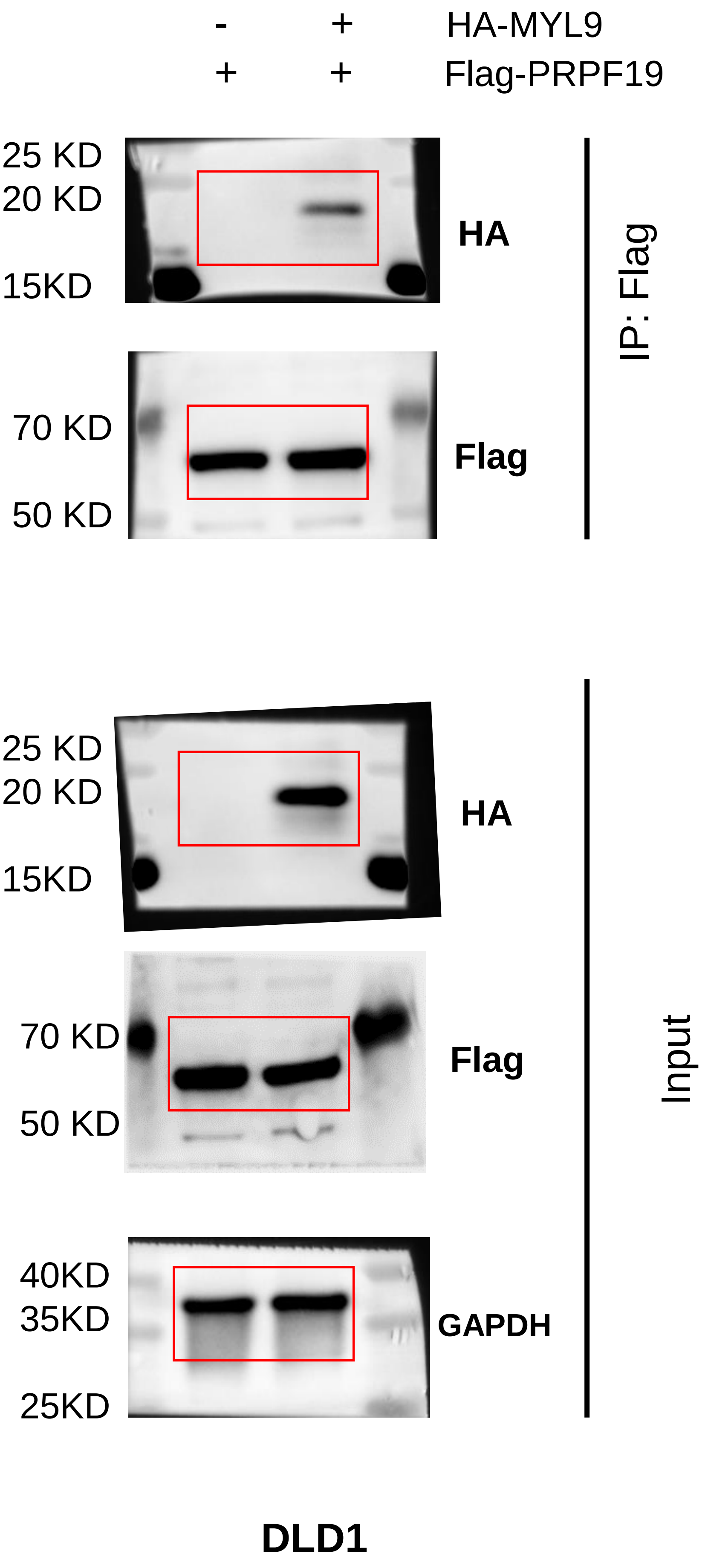
- + HA-MYL9
+ + Flag-PRPF19
25 KD
20 KD
15KD
HA
70 KD
50 KD
Flag
IP: Flag
25 KD
20 KD
15KD
HA
70 KD
50 KD
Flag
40KD
35KD
25KD
GAPDH
Input
DLD1

Figure 3B

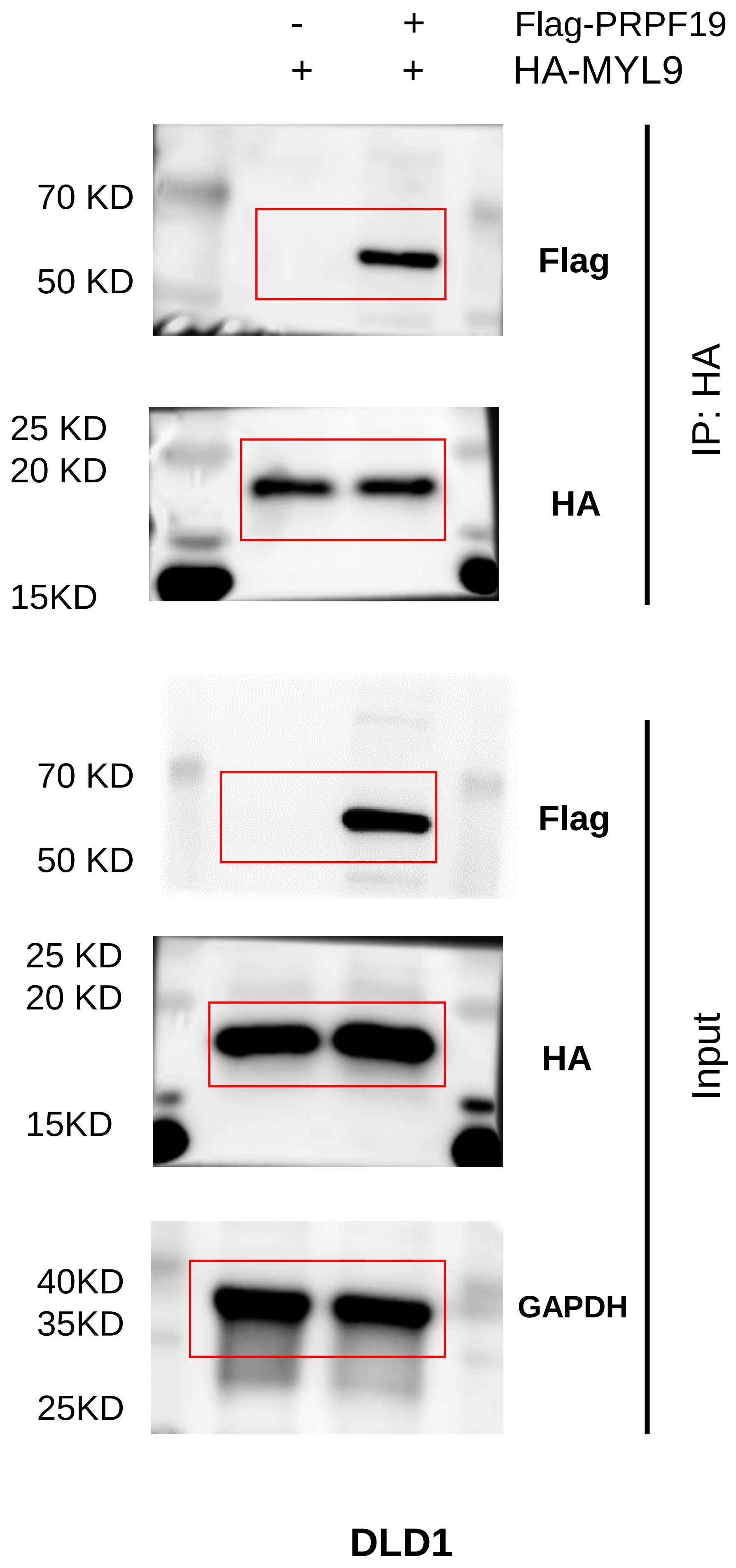

- + Flag-PRPF19
+ + HA-MYL9
70 KD
50 KD
Flag
25 KD
20 KD
15KD
HA
IP: HA
70 KD
50 KD
Flag
25 KD
20 KD
15KD
HA
40KD
35KD
25KD
GAPDH
Input
DLD1

Figure 3C

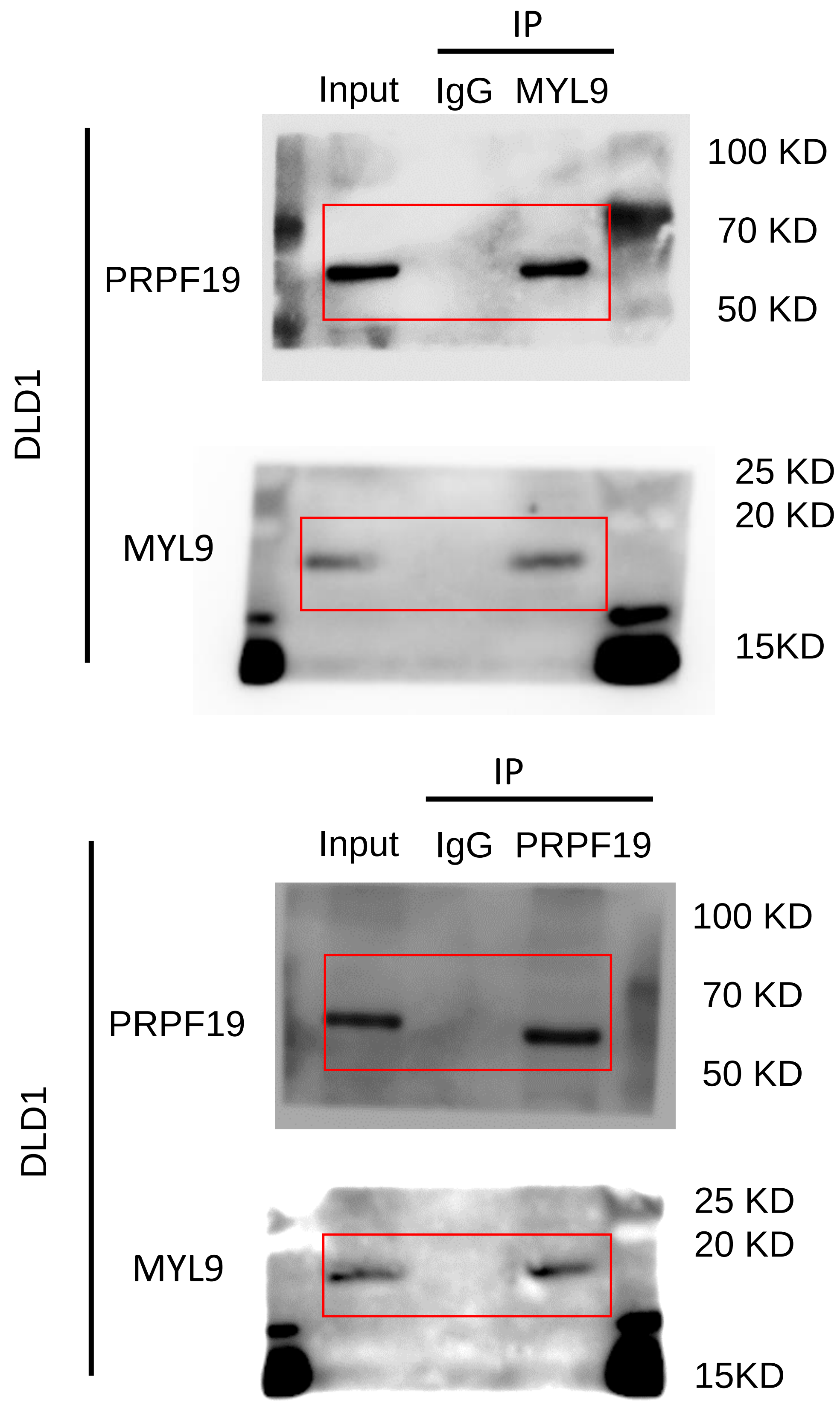
IP
Input
IgG
MYL9
DLD1
PRPF19
MYL9
100 KD
70 KD
50 KD
25 KD
20 KD
15KD
IP
Input
IgG
PRPF19
DLD1
PRPF19
MYL9
100 KD
70 KD
50 KD
25 KD
20 KD
15KD

Figure 3E

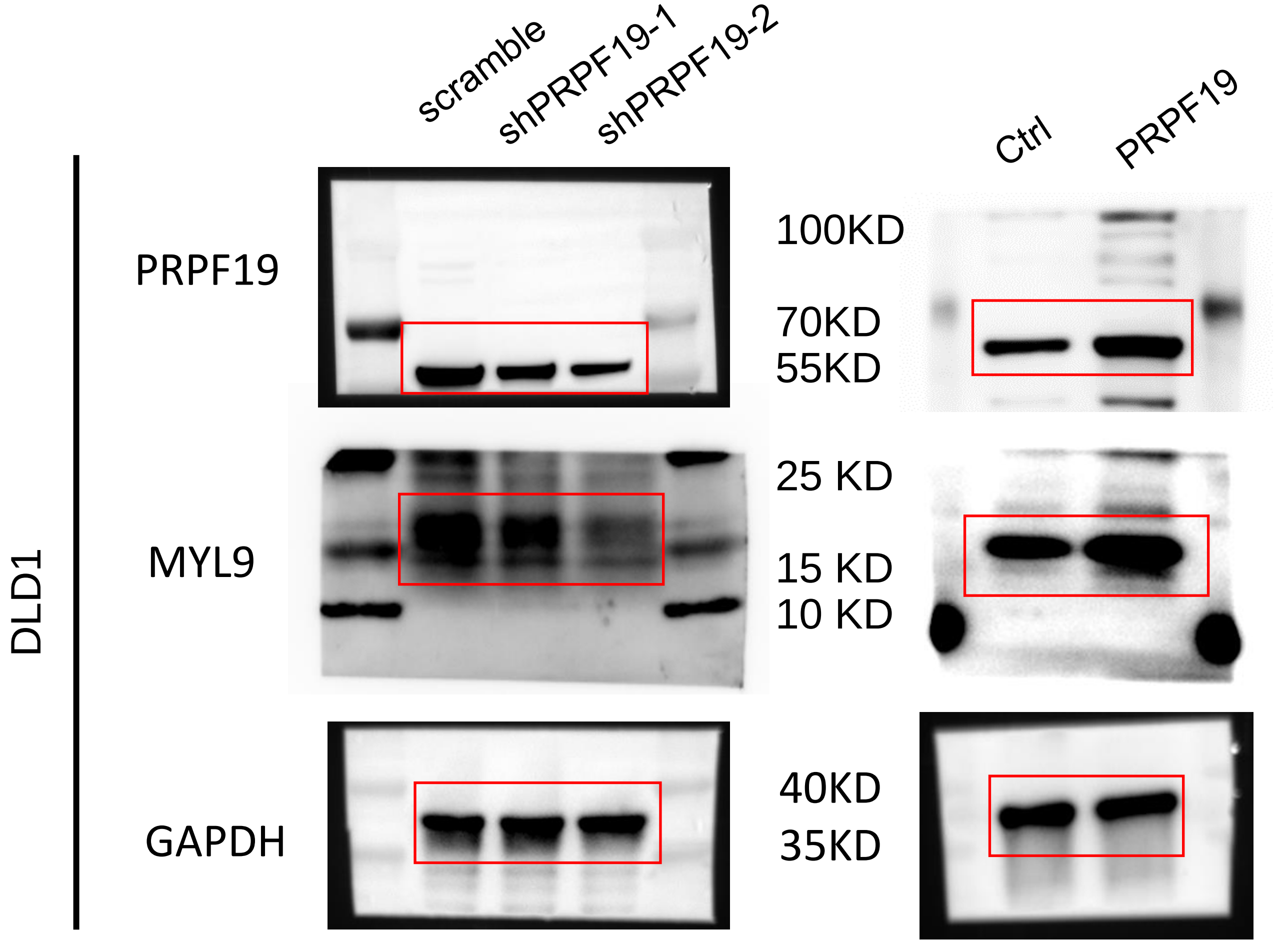

scramble
shPRPF19-1
shPRPF19-2
Ctrl
PRPF19
DLD1
PRPF19
MYL9
GAPDH
100KD
70KD
55KD
25 KD
15 KD
10 KD
40KD
35KD

Figure 3F

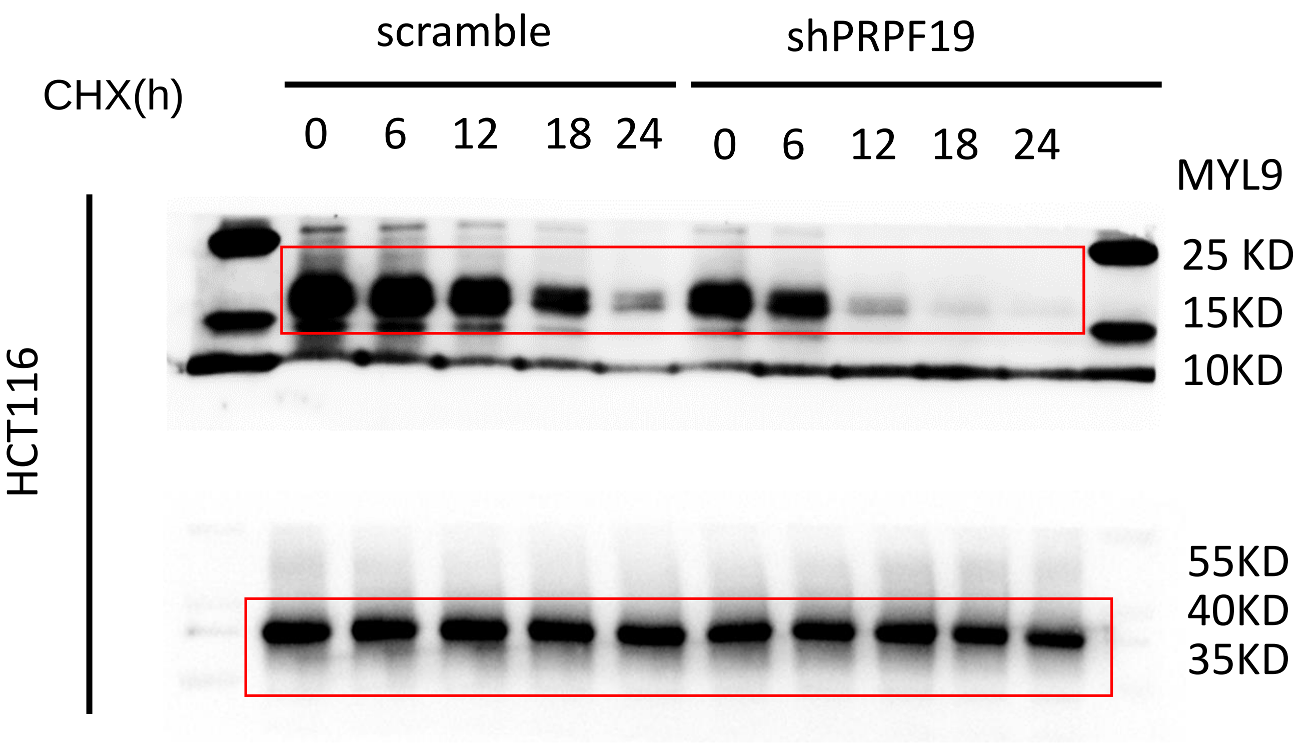
scramble
shPRPF19
CHX(h)
0
6
12
18
24
0
6
12
18
24
MYL9
25 KD
15KD
10KD
HCT116
55KD
40KD
35KD

Figure 3G

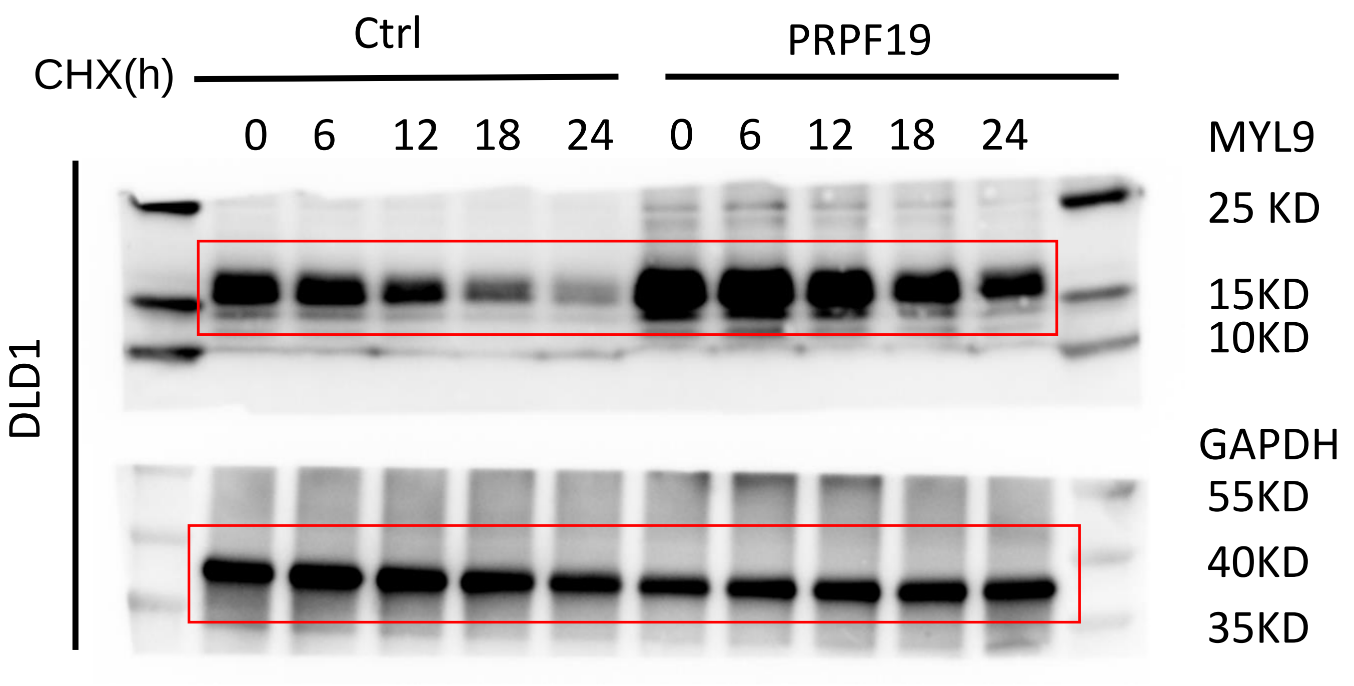
Ctrl
PRPF19
CHX(h)
0 6 12 18 24 0 6 12 18 24
MYL9
25 KD
15KD
10KD
DLD1
GAPDH
55KD
40KD
35KD

Figure 4A

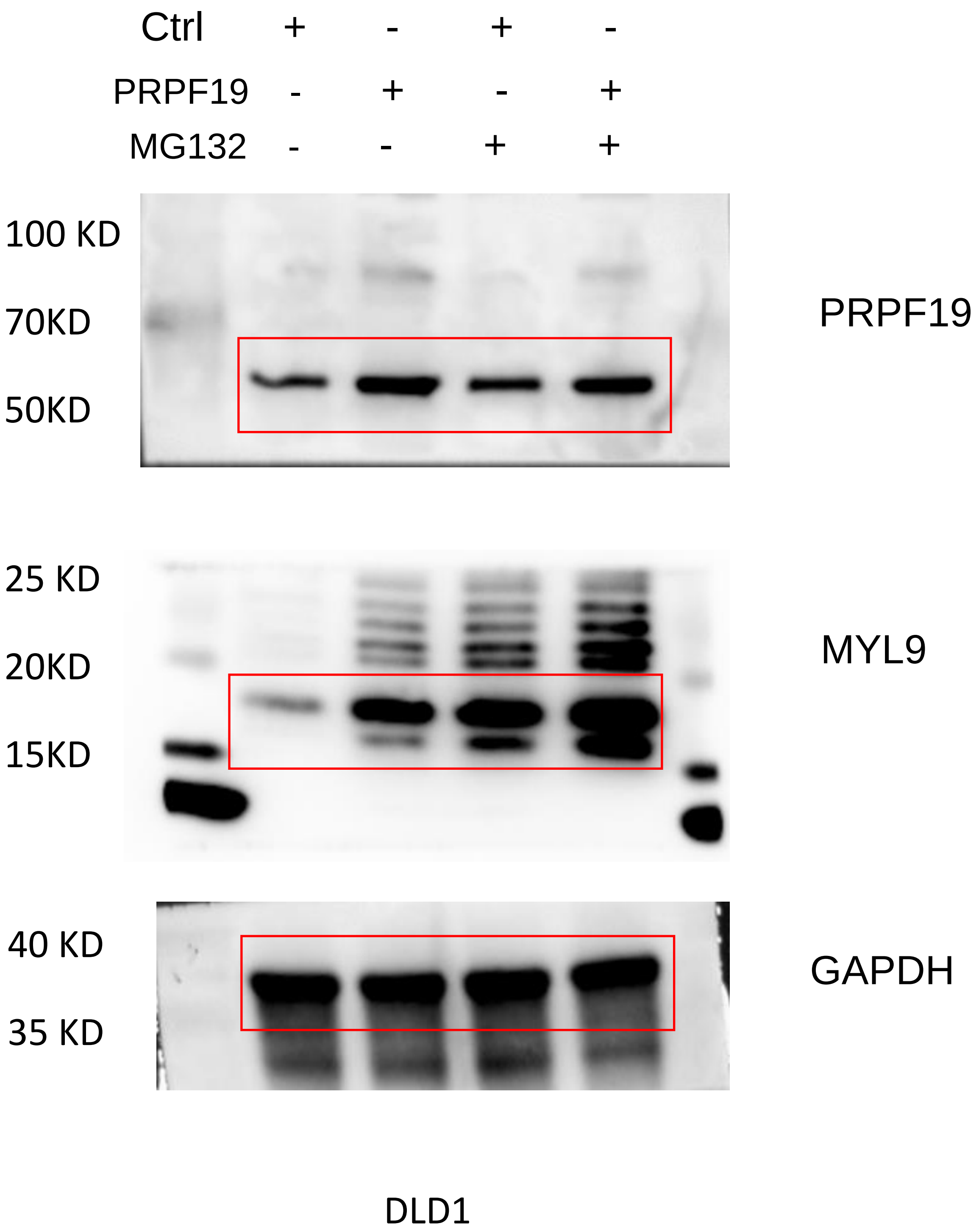

Ctrl + - + -
PRPF19 - + - +
MG132 - - + +
100 KD
70KD
50KD
PRPF19
25 KD
20KD
15KD
MYL9
40 KD
35 KD
GAPDH
DLD1

Figure 4B

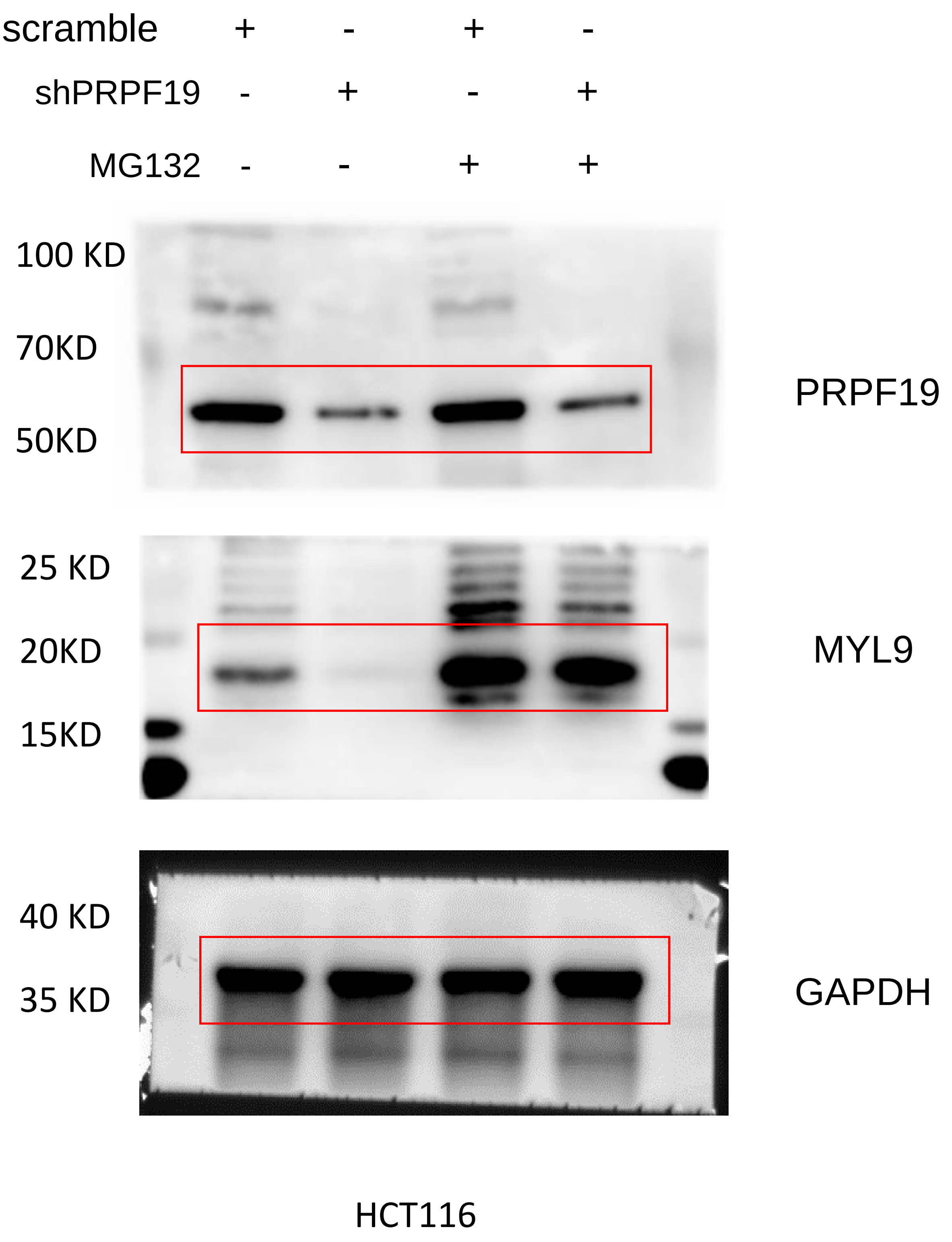
scramble + - + -
shPRPF19 - + - +
MG132 - - + +
100 KD
70KD
50KD
PRPF19
25 KD
20KD
15KD
MYL9
40 KD
35 KD
GAPDH
HCT116

Figure 4C

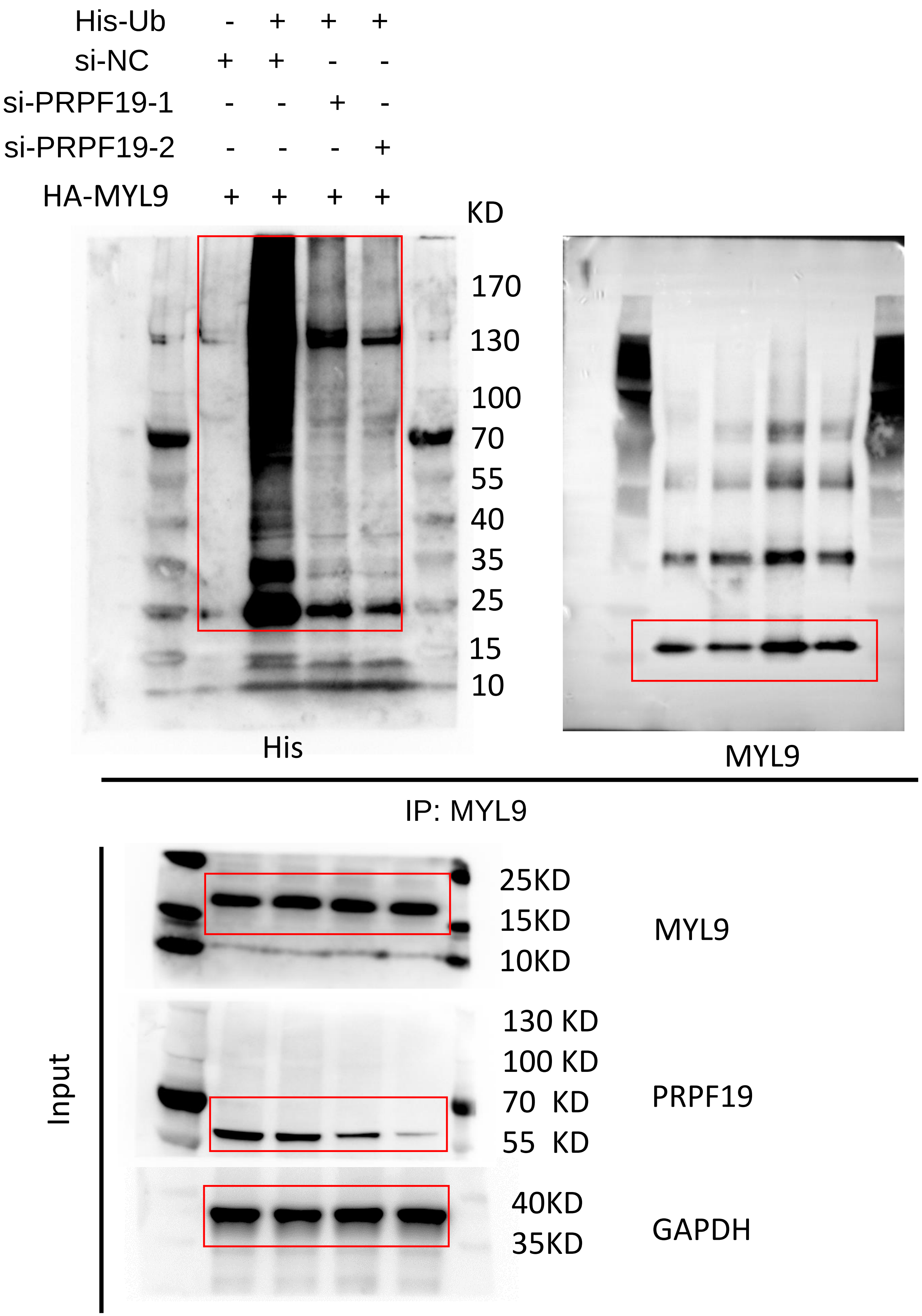

His-Ub - + + +
si-NC + + - -
si-PRPF19-1 - - + -
si-PRPF19-2 - - - +
HA-MYL9 + + + +
KD
170
130
100
70
55
40
35
25
15
10
His
MYL9
IP: MYL9
Input
25KD
15KD
10KD
MYL9
130 KD
100 KD
70 KD
55 KD
PRPF19
40KD
35KD
GAPDH

Figure 4D

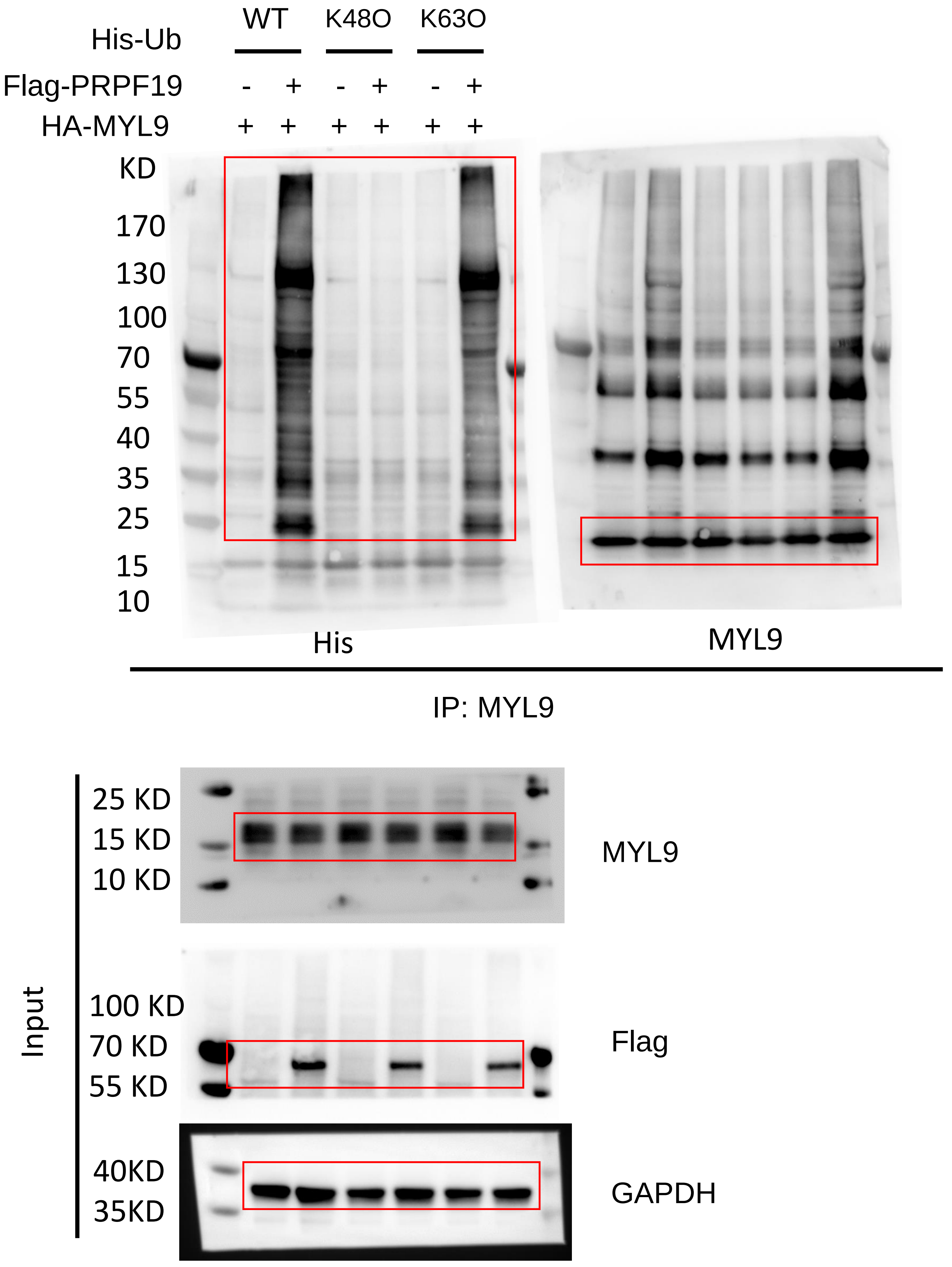
His-Ub
WT
K48O
K63O
Flag-PRPF19
- + - + - +
HA-MYL9
+ + + + + +
KD
170
130
100
70
55
40
35
25
15
10
His
MYL9
IP: MYL9
Input
25 KD
15 KD
10 KD
MYL9
100 KD
70 KD
55 KD
Flag
40KD
35KD
GAPDH

Figure 4E

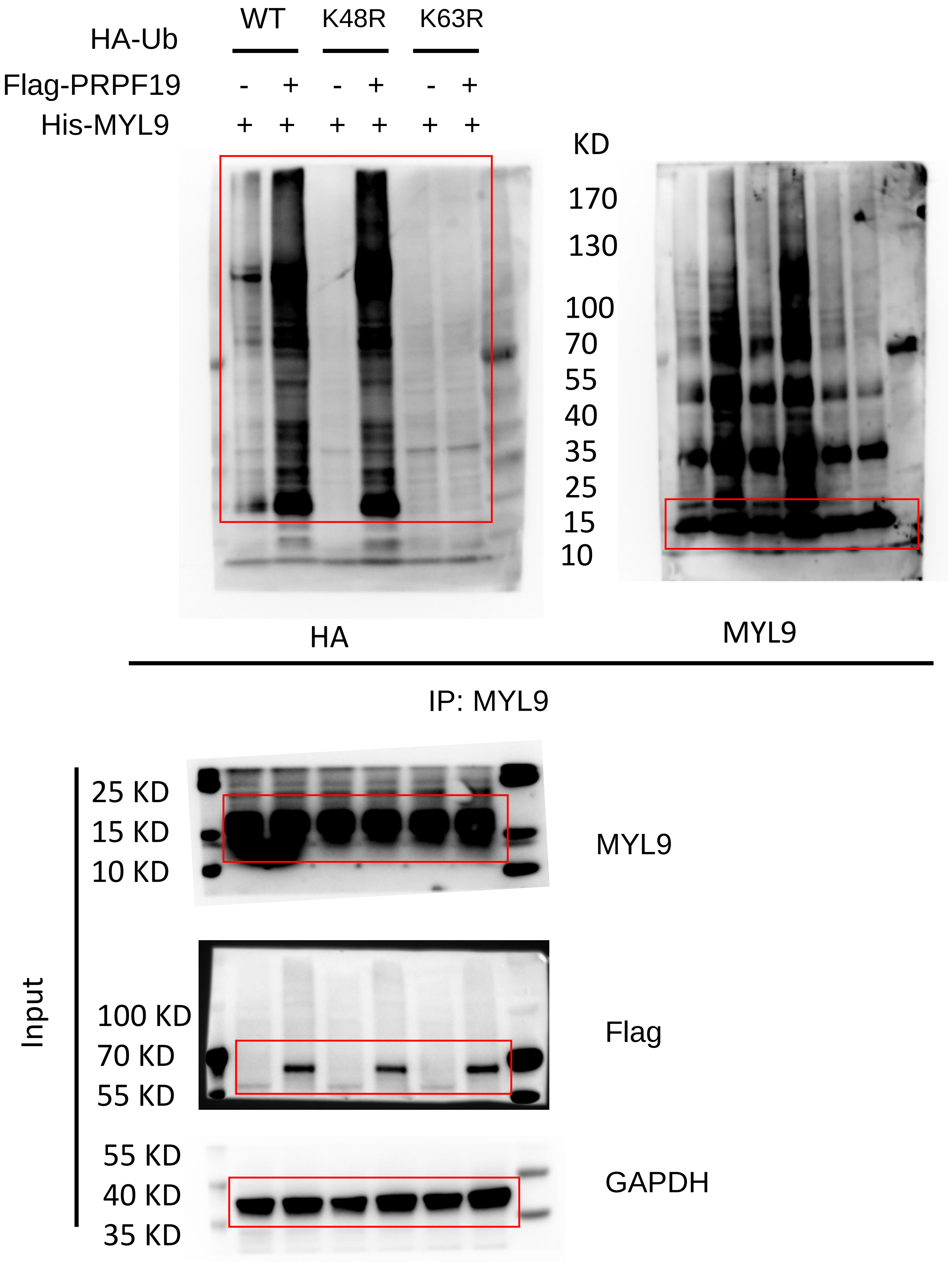
HA-Ub
WT
K48R
K63R
Flag-PRPF19
- + - + - +
His-MYL9
+ + + + + +
KD
170
130
100
70
55
40
35
25
15
10
HA
MYL9
IP: MYL9
Input
25 KD
15 KD
10 KD
MYL9
100 KD
70 KD
55 KD
Flag
55 KD
40 KD
35 KD
GAPDH

Figure 4F

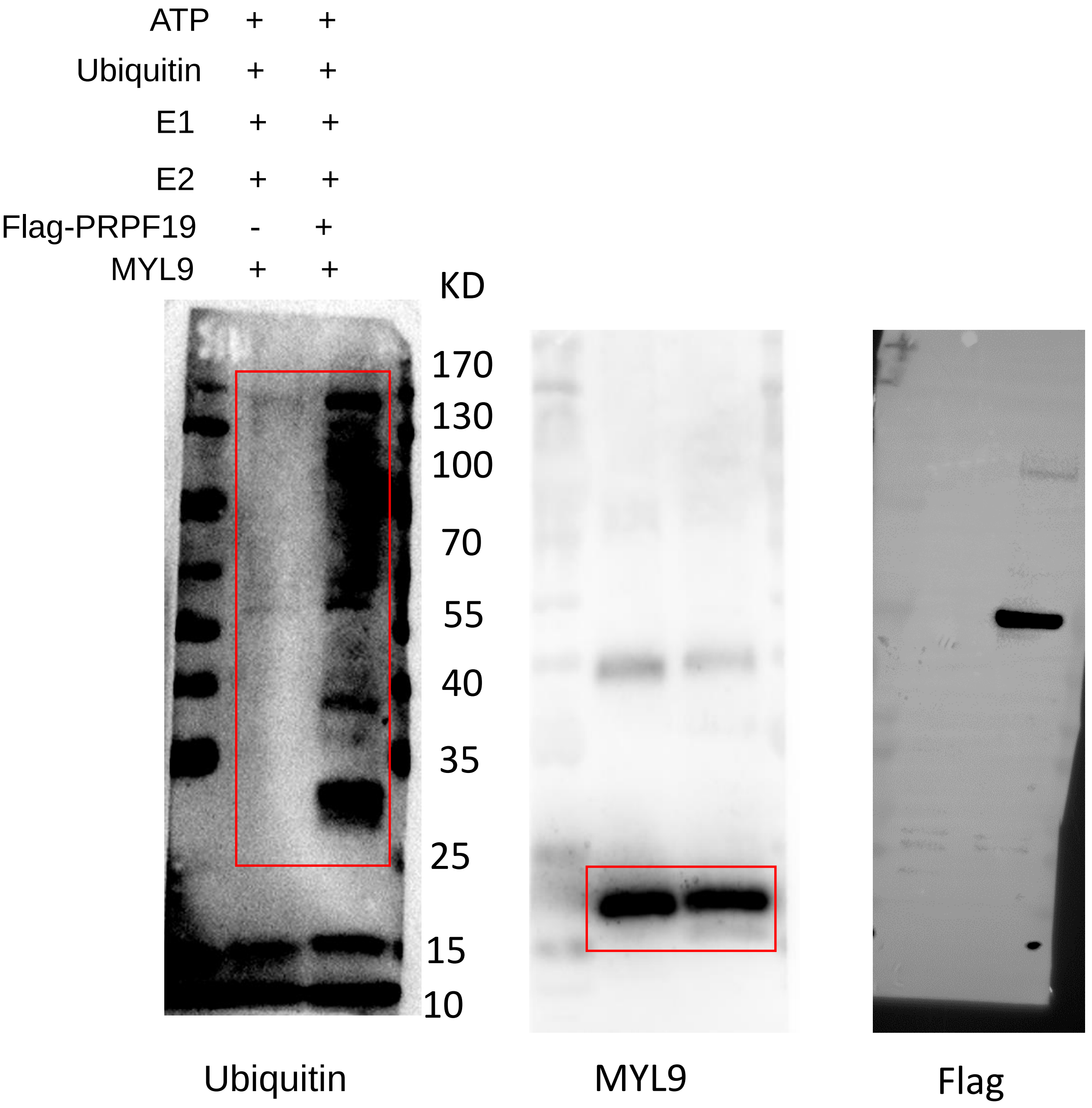

ATP + +
Ubiquitin + +
E1 + +
E2 + +
Flag-PRPF19 - +
MYL9 + +
KD
170
130
100
70
55
40
35
25
15
10
Ubiquitin
MYL9
Flag

Figure 6C

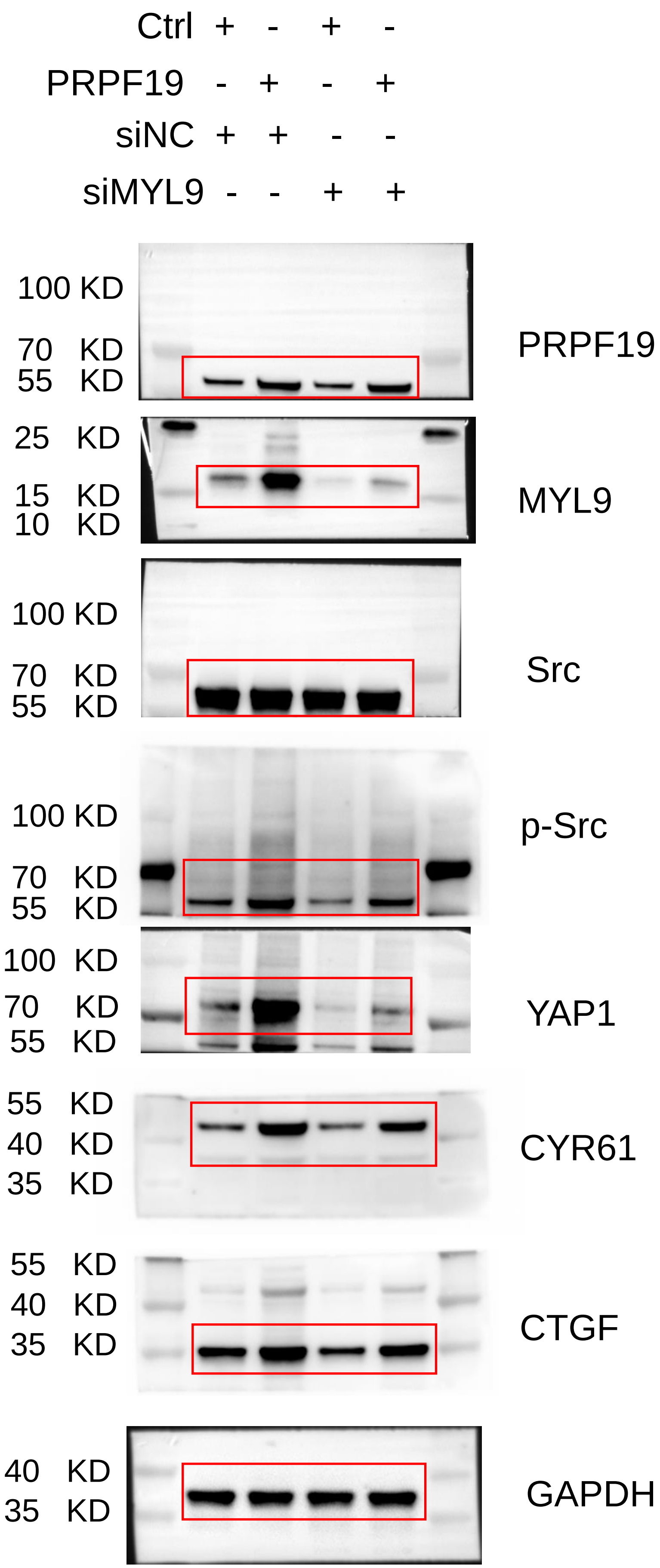

Ctrl + - + -
PRPF19 - + - +
siNC + + - -
siMYL9 - - + +
100 KD
70 KD
55 KD
PRPF19
25 KD
15 KD
10 KD
MYL9
100 KD
70 KD
55 KD
Src
100 KD
70 KD
55 KD
p-Src
100 KD
70 KD
55 KD
YAP1
55 KD
40 KD
35 KD
CYR61
55 KD
40 KD
35 KD
CTGF
40 KD
35 KD
GAPDH

Figure 6D

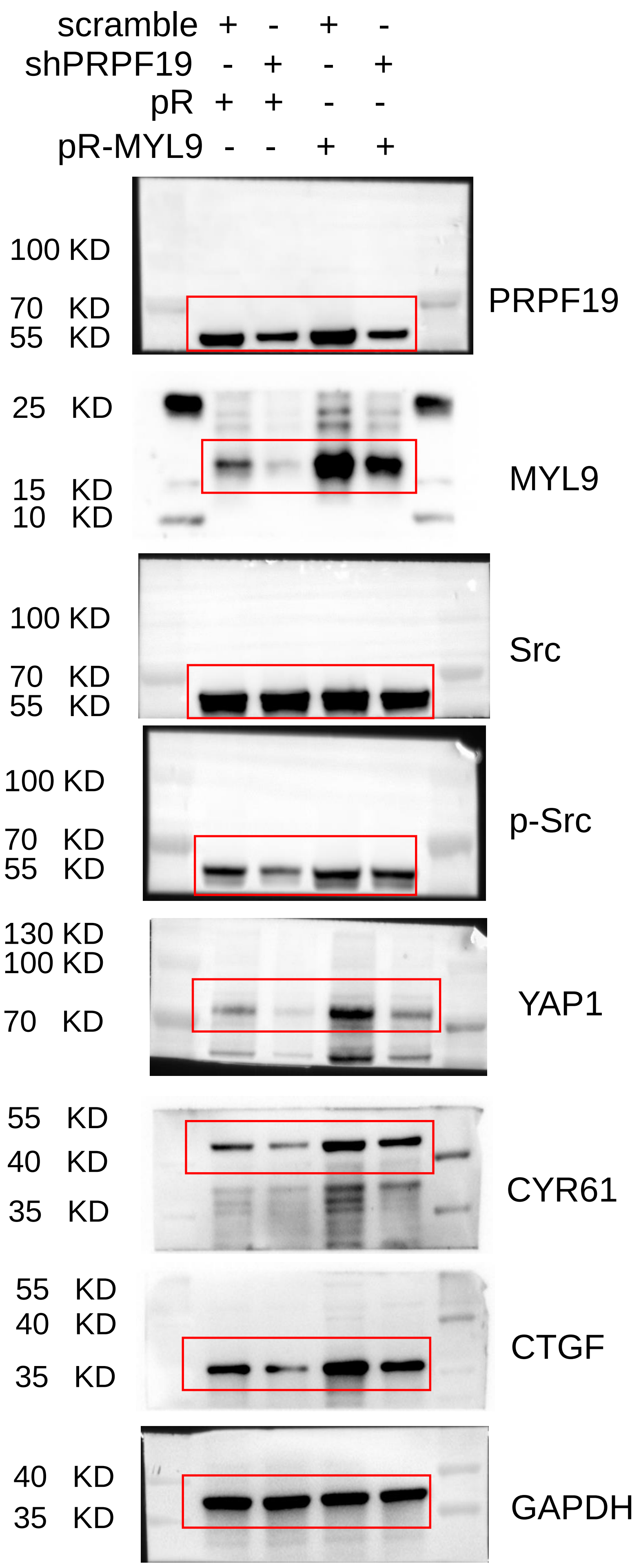
scramble + - + -
shPRPF19 - + - +
pR + + - -
pR-MYL9 - - + +
100 KD
70 KD
55 KD
PRPF19
25 KD
15 KD
10 KD
MYL9
100 KD
70 KD
55 KD
Src
100 KD
70 KD
55 KD
p-Src
130 KD
100 KD
70 KD
YAP1
55 KD
40 KD
35 KD
CYR61
55 KD
40 KD
35 KD
CTGF
40 KD
35 KD
GAPDH

Supplementary Figure 1C

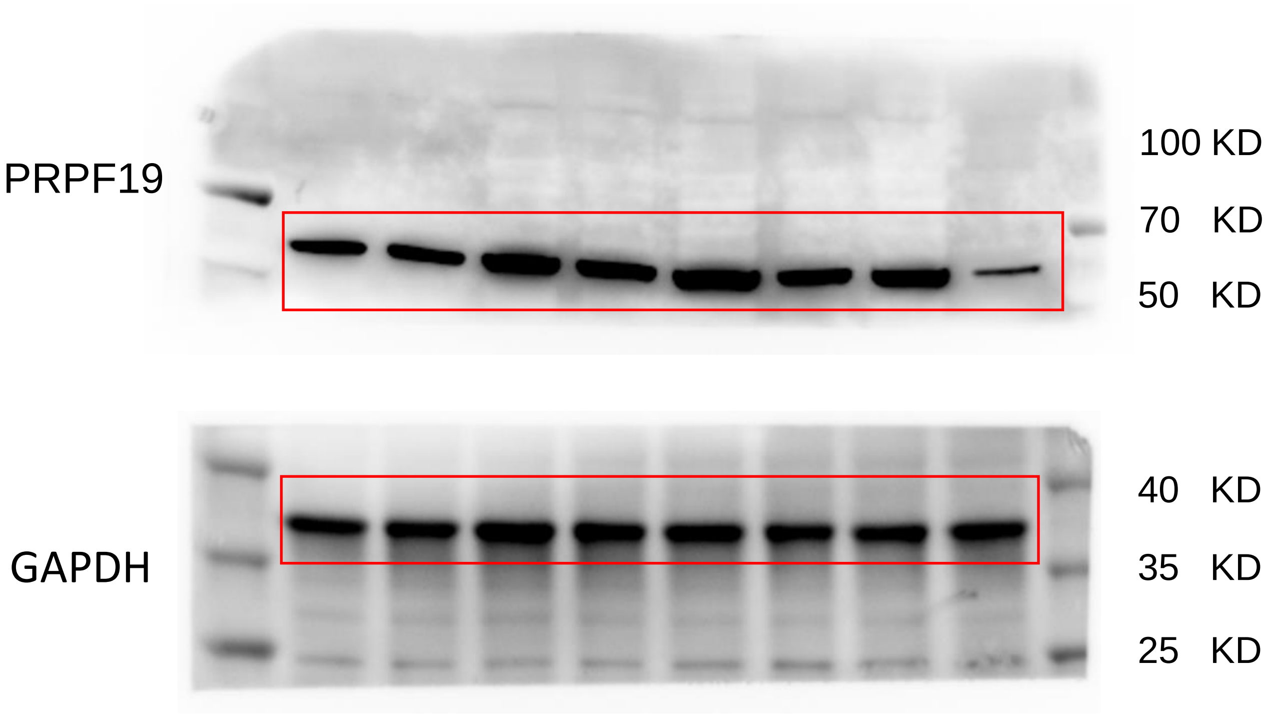

Supplementary Figure 3A

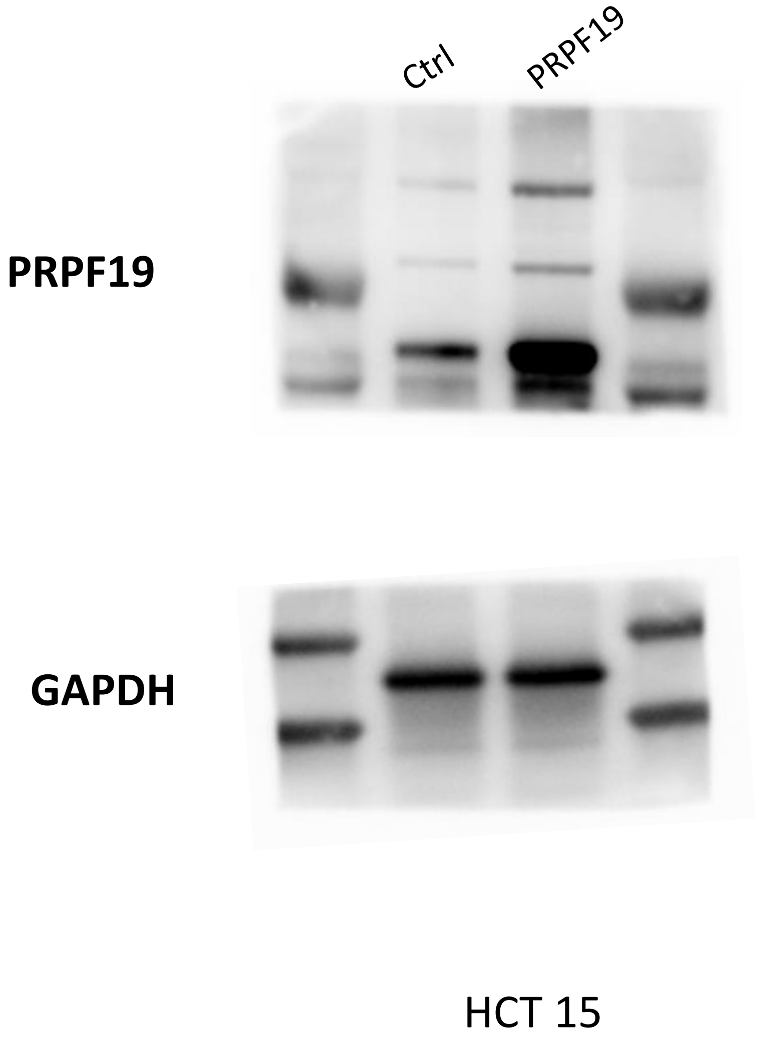

Supplementary Figure 5A

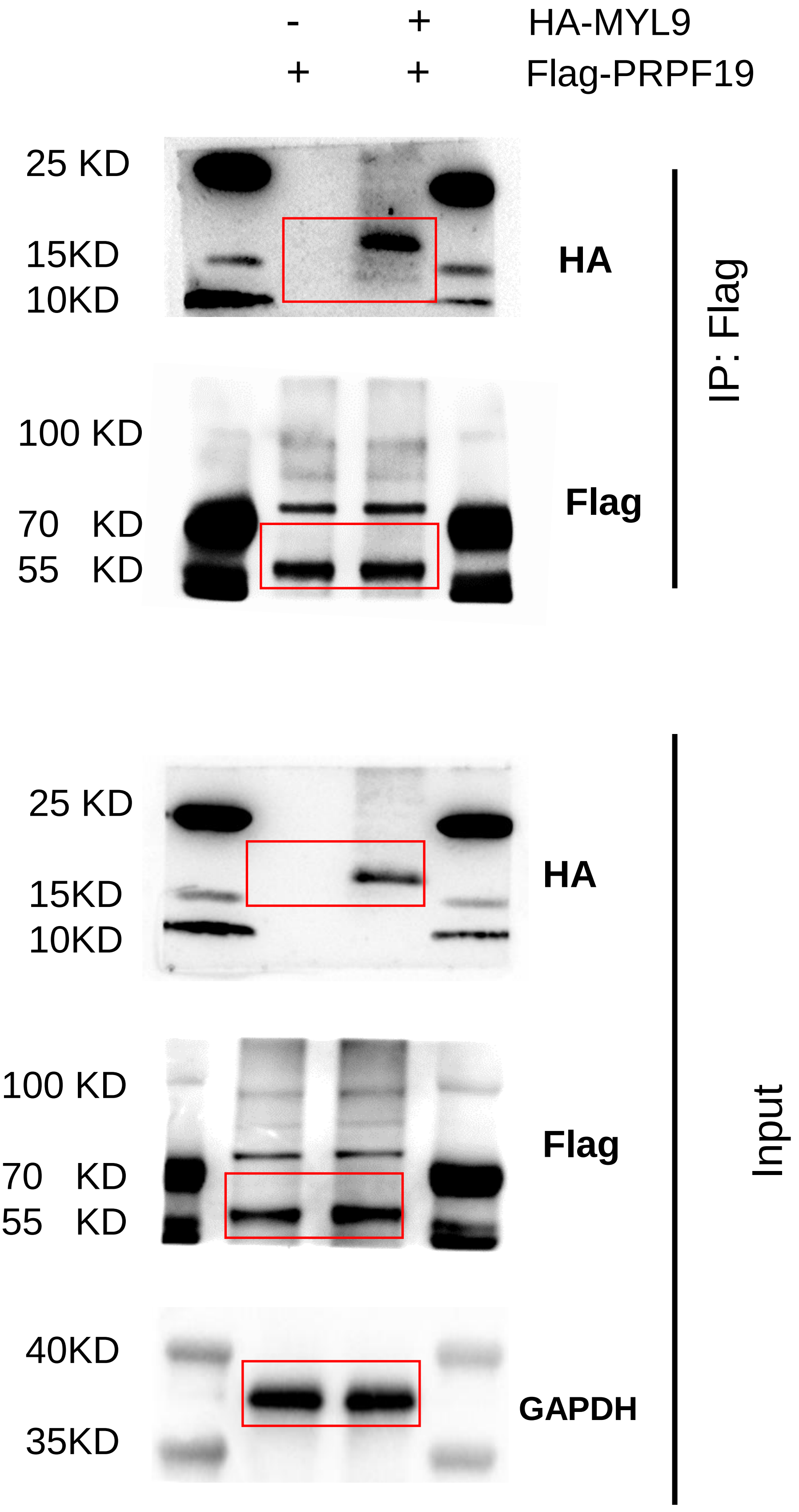

HCT15

Supplementary Figure 5A

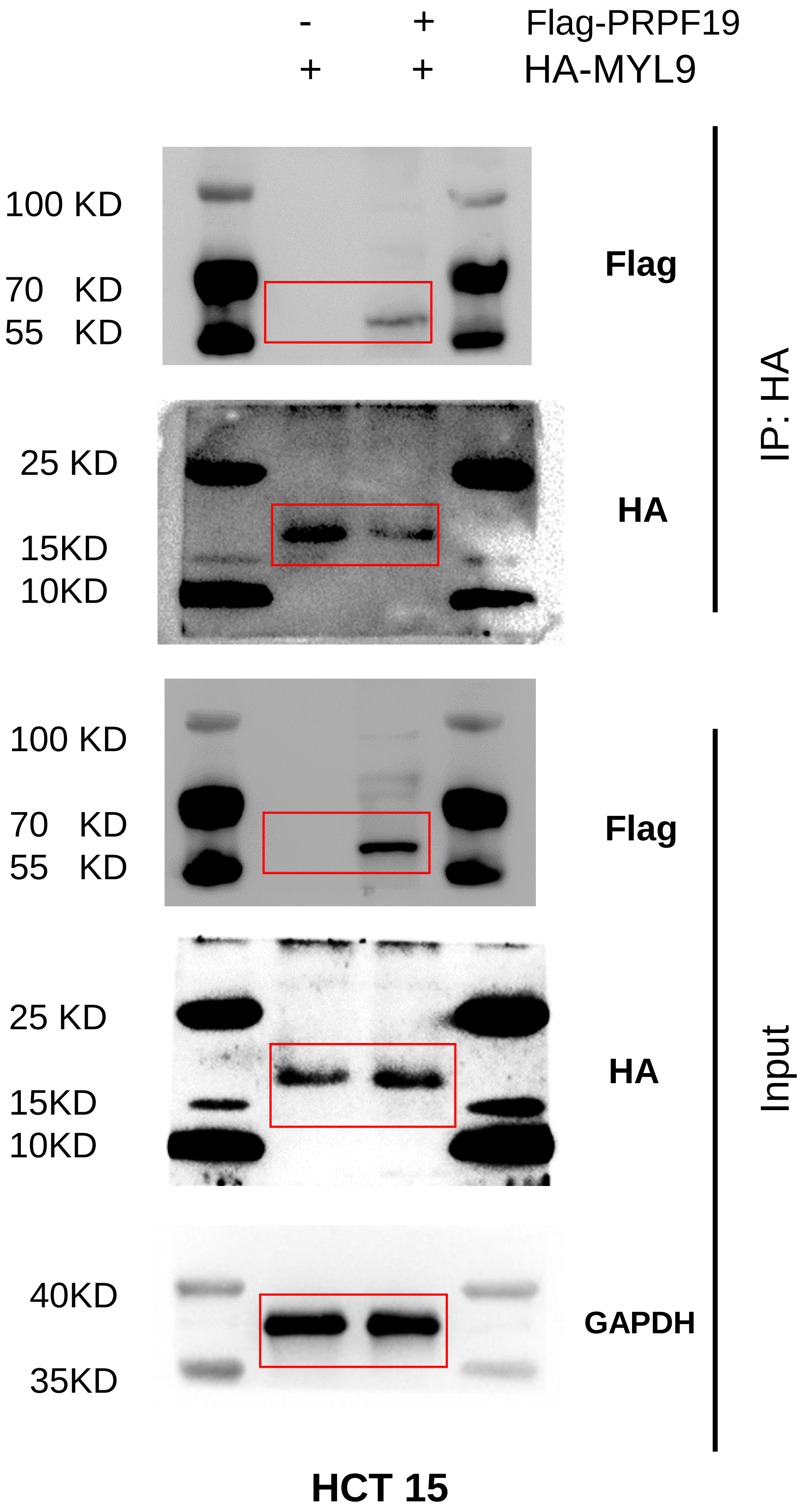

Supplementary Figure 5B

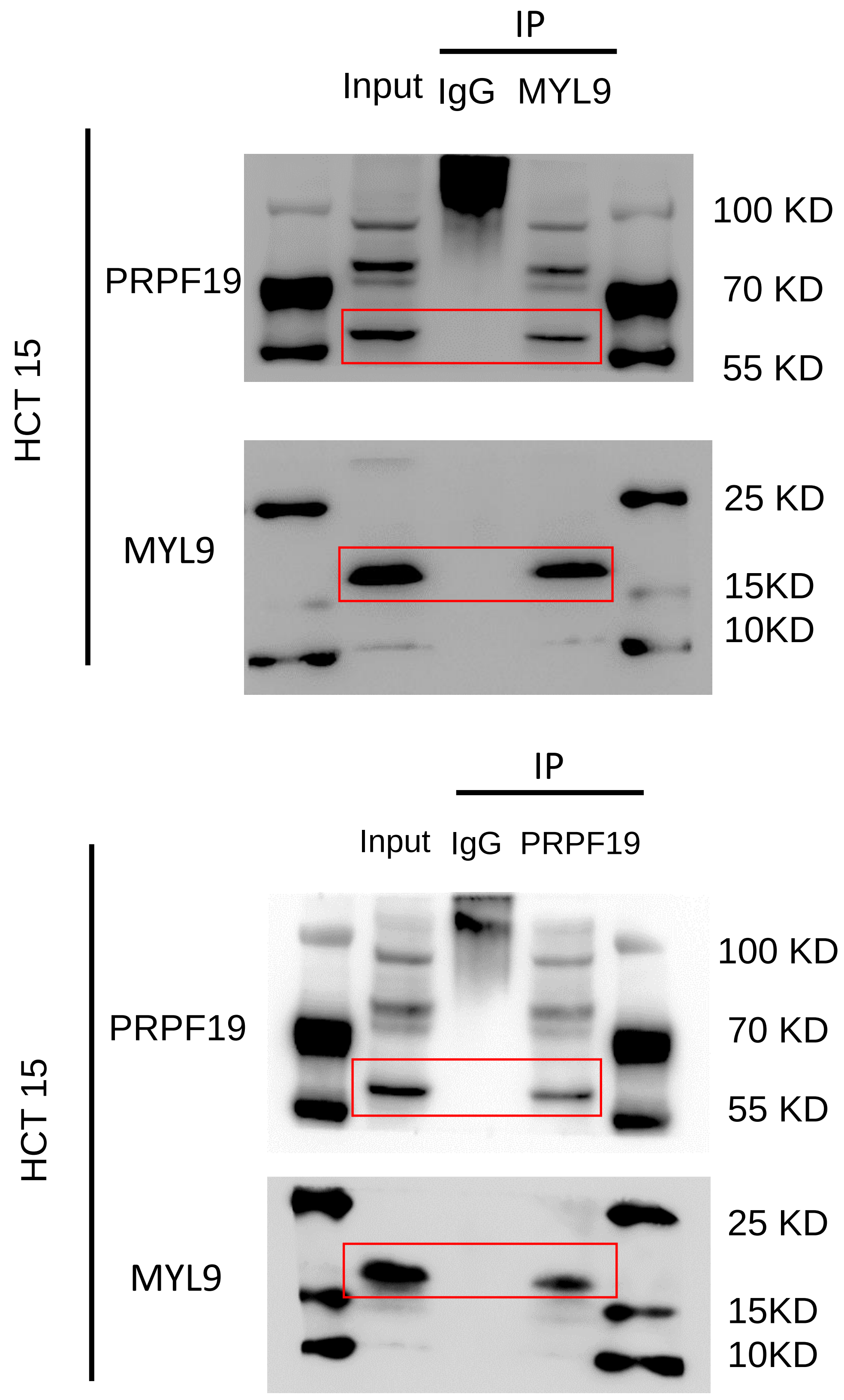

Supplementary Figure 5C

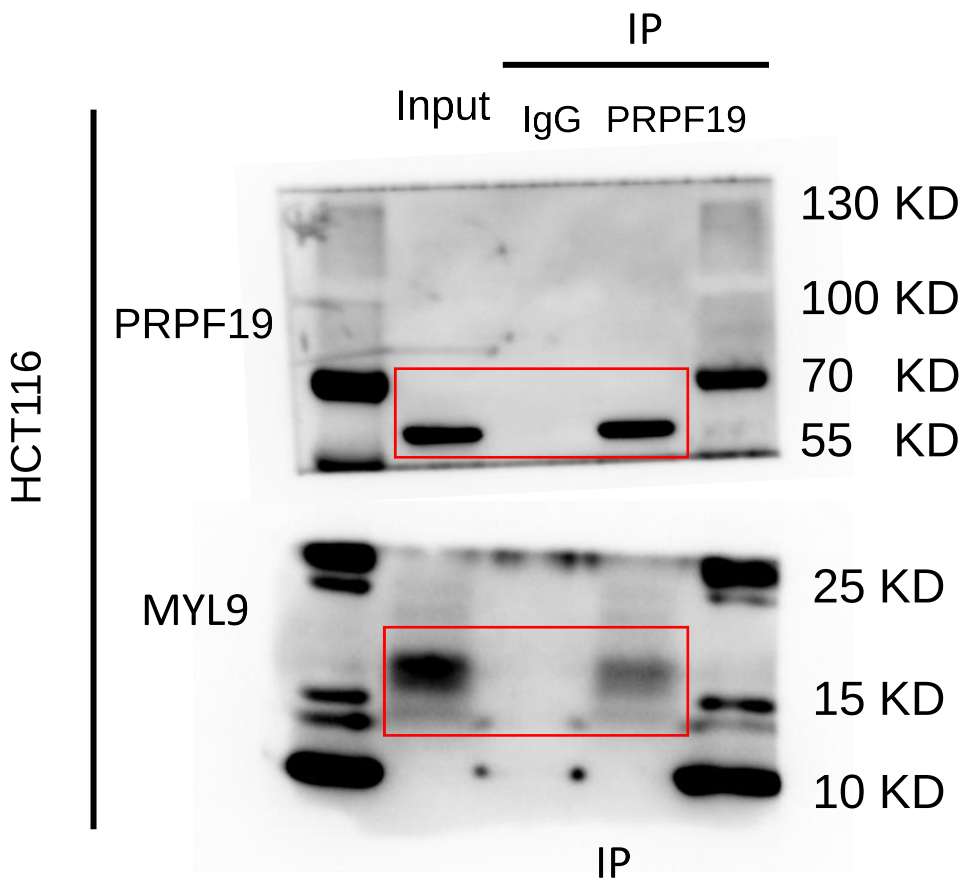

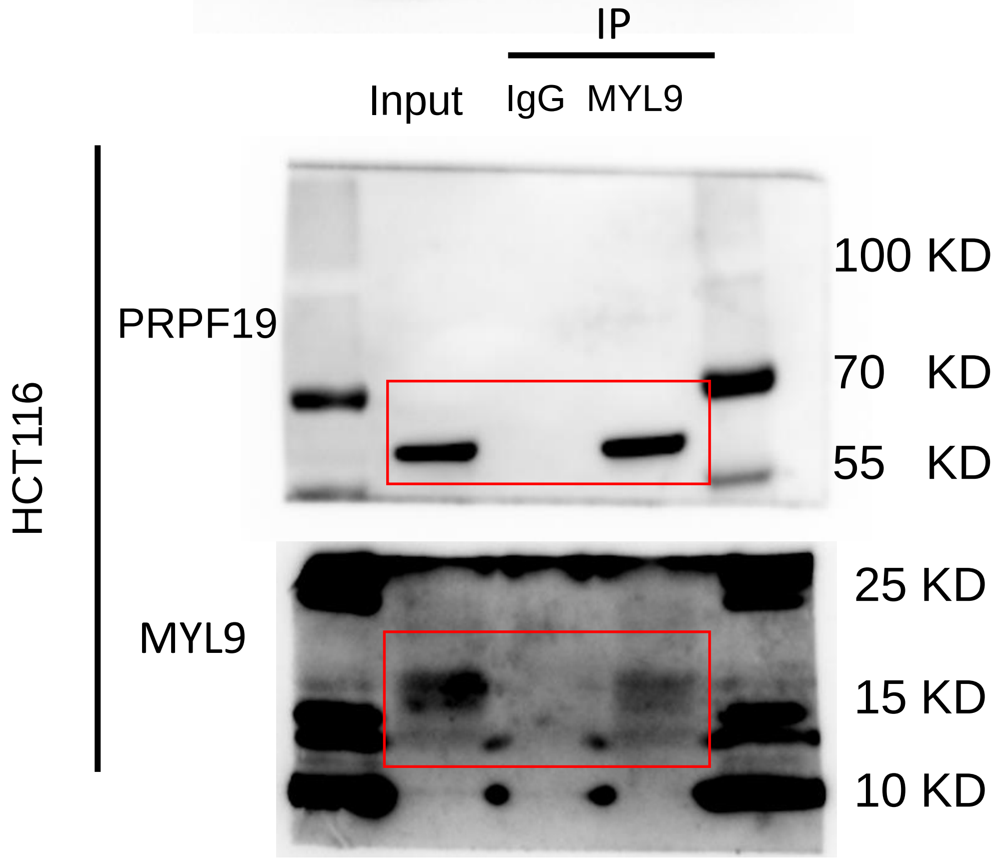

Supplementary Figure 5D

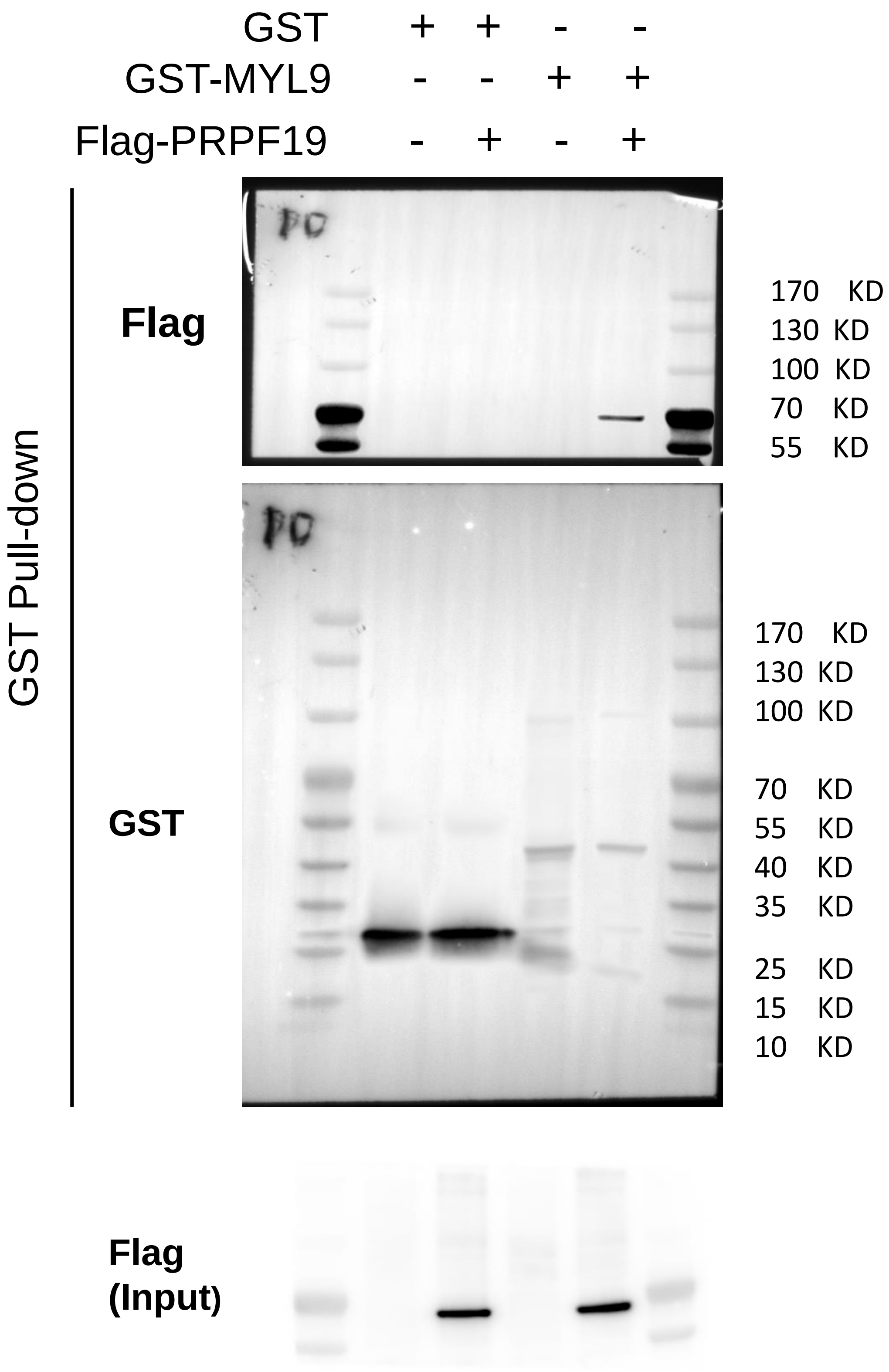

Supplementary Figure 5F

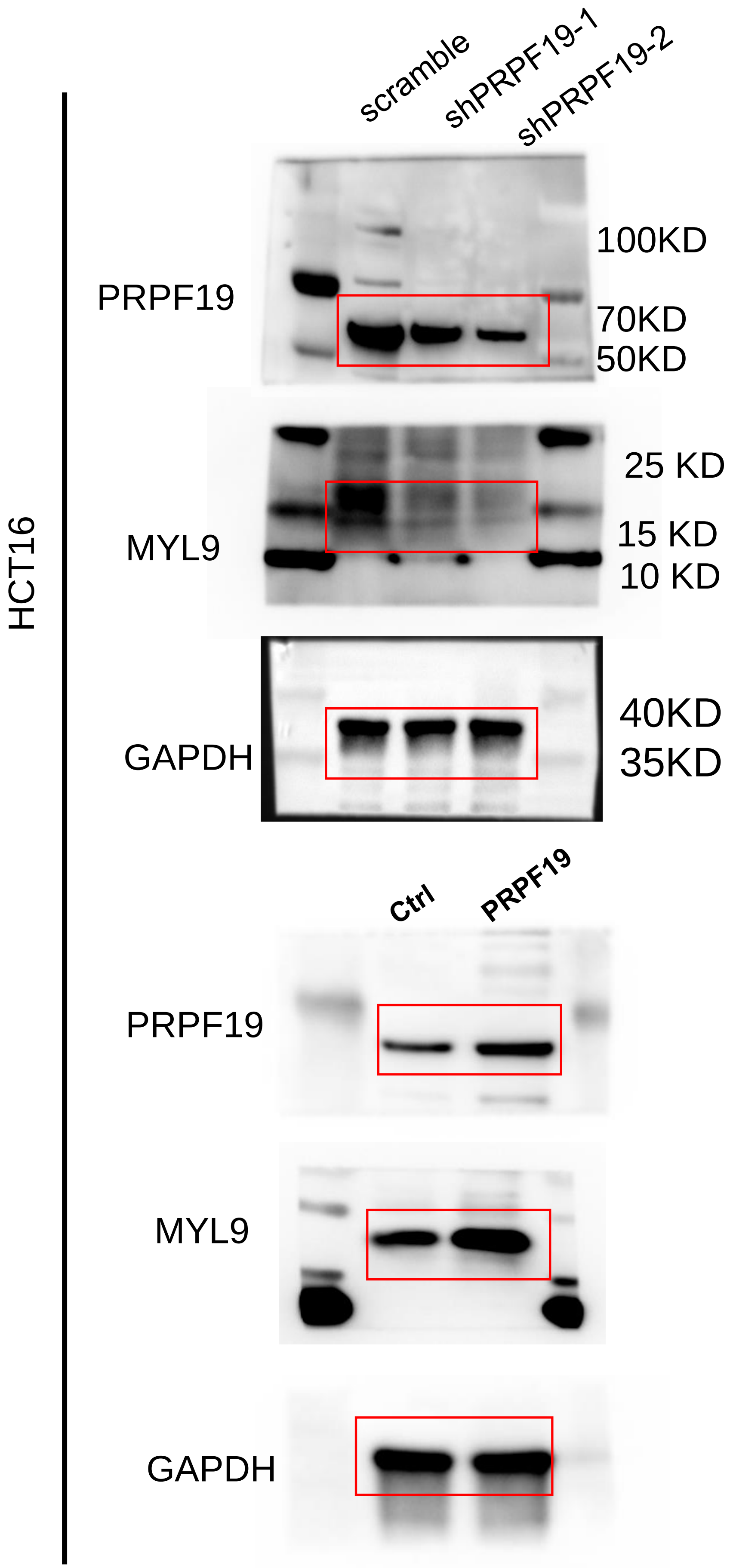

Supplementary Figure 5H

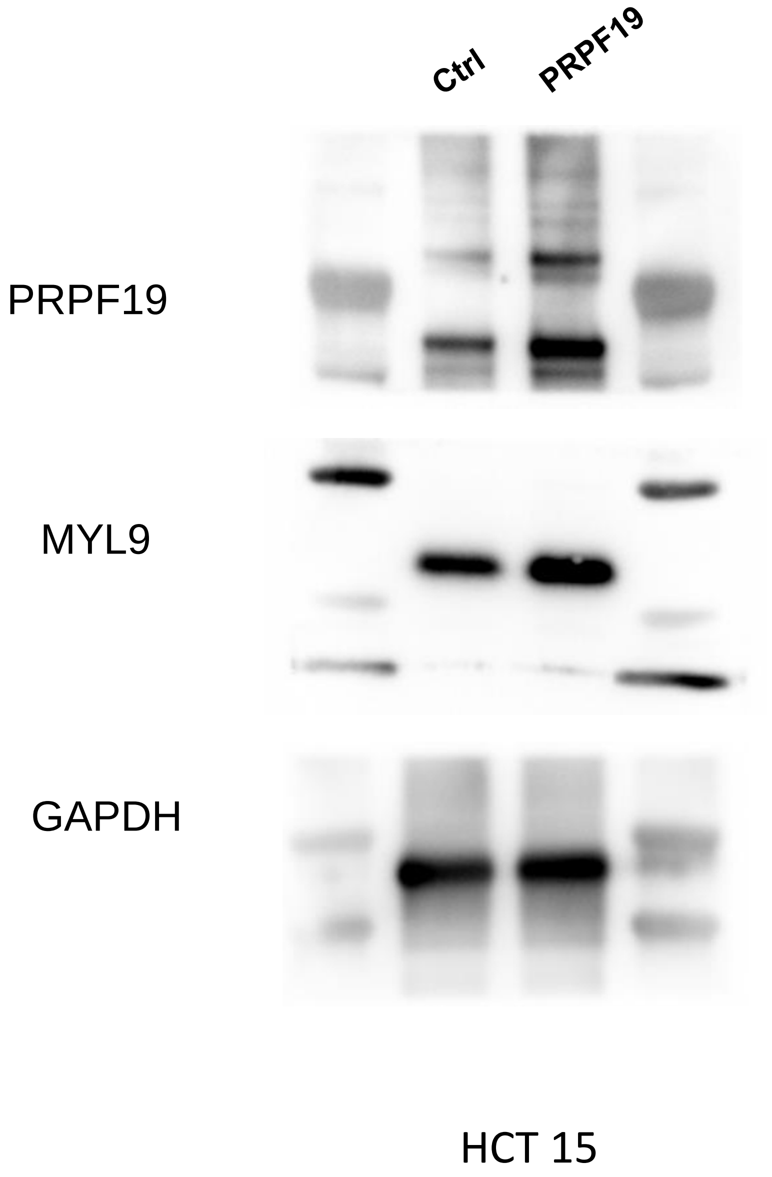

Supplementary Figure 6A

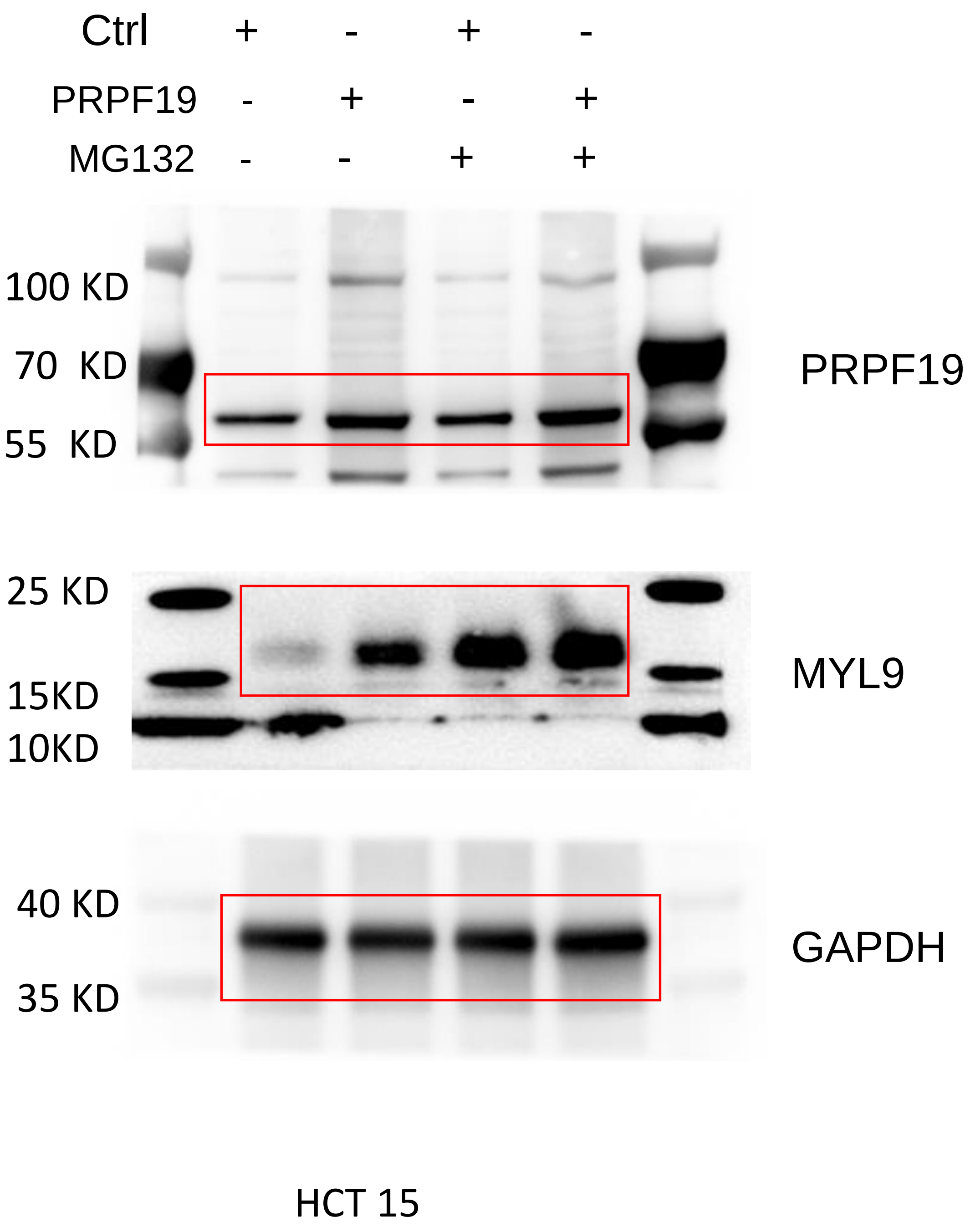

Supplement: Supplementary file 13 — Original Data File [file 41419_2023_5776_MOESM13_ESM.pdf]
